# Modulating the Properties of Fe(III) Macrocyclic MRI Contrast Agents by Appending Sulfonate or Hydroxyl Groups

**DOI:** 10.3390/molecules25102291

**Published:** 2020-05-13

**Authors:** Didar Asik, Rachel Smolinski, Samira M. Abozeid, Travis B. Mitchell, Steven G. Turowski, Joseph A. Spernyak, Janet R. Morrow

**Affiliations:** 1 Department of Chemistry, University at Buffalo, The State University of New York, Amherst, NY 14260, USA; didarasi@buffalo.edu (D.A.); resmolin@buffalo.edu (R.S.); samiramo@buffalo.edu (S.M.A.); travismi@buffalo.edu (T.B.M.); 2 Department of Cell Stress Biology, Roswell Park Comprehensive Cancer Center, Buffalo, NY 14263, USA; steven.turowski@roswellpark.org (S.G.T.); Joseph.Spernyak@roswellpark.org (J.A.S.)

**Keywords:** Fe(III) complexes, T_1_ contrast agents, MRI, macrocyclic ligands, TACN, sulfonate group, pharmacokinetics, in vivo, kidney image, relaxivity

## Abstract

Complexes of Fe(III) that contain a triazacyclononane (TACN) macrocycle, two pendant hydroxyl groups, and a third ancillary pendant show promise as MRI contrast agents. The ancillary group plays an important role in tuning the solution relaxivity of the Fe(III) complex and leads to large changes in MRI contrast enhancement in mice. Two new Fe(III) complexes, one with a third coordinating hydroxypropyl pendant, Fe(L2), and one with an anionic non-coordinating sulfonate group, Fe(L1)(OH_2_), are compared. Both complexes have a deprotonated hydroxyl group at neutral pH and electrode potentials representative of a stabilized trivalent iron center. The r_1_ relaxivity of the Fe(L1)(OH_2_) complex is double that of the saturated complex, Fe(L2), at 4.7 T, 37 °C in buffered solutions. However, variable-temperature ^17^O-NMR experiments show that the inner-sphere water of Fe(L1)(OH_2_) does not exchange rapidly with bulk water under these conditions. The pendant sulfonate group in Fe(L1)(OH_2_) confers high solubility to the complex in comparison to Fe(L2) or previously studied analogues with benzyl groups. Dynamic MRI studies of the two complexes showed major differences in their pharmacokinetics clearance rates compared to an analogue containing a benzyl ancillary group. Rapid blood clearance and poor binding to serum albumin identify Fe(L1)(OH_2_) for development as an extracellular fluid contrast agent.

## 1. Introduction 

Nearly 40% of clinical MRI procedures use a contrast agent to produce enhanced images, and nearly all contrast agents are Gd(III) coordination complexes. Although Gd-based contrast agents are highly successful, there are concerns about potential toxicity and retention from long-term exposure, particularly for patients with renal insufficiency [1,2,3,4,5,6,7]. These concerns have motivated research into alternatives for Gd that utilize transition metal ions. Potential candidates include high-spin (S = 5/2) Mn(II) and Fe(III) complexes in order to capitalize on their large effective magnetic moments and long electronic relaxation times [7,8]. These two metal ions are the most promising for the development of Gd-free MRI contrast agents, based on their magnetic properties and their roles as trace elements in human biology. There has been much progress in the development of Mn(II) complexes as MRI probes [9,10,11,12,13]. However, despite the prominent role of iron in human biology, there are significantly fewer studies on Fe(III)-based contrast agents [14,15,16,17,18,19,20].

The Fe(III) complexes that were studied in the early years of MRI contrast agent development were not very effective in comparison to those of Gd(III) [21]. In many cases, similar donor groups and chelate frameworks were used despite the difference in the coordination chemistry of the two ions [15,17,22,23,24]. For example, the ionic radius of Fe(III) (0.785 A for six-coordinate, high spin) is much smaller than that of Gd(III) (1.25 A for nine-coordinate) [25]. The small radius of Fe(III) complexes leads to predominantly six-coordinate and some seven-coordinate complexes, and ligand design should reflect these preferences. Moreover, Fe(III) is a much stronger Lewis acid than either Gd(III) or Mn(II) and forms robust complexes that are relatively inert to dissociation. 

A major challenge in the development of iron agents is the difficulty of controlling the coordination chemistry associated with the highly polarizing Fe(III) center [26]. For example, Fe(III) complexes with coordination sites for water ligands may instead form hydroxide complexes or dimeric complexes with bridging oxides [27,28,29,30,31]. Fe(III) complexes with hexadentate polyaminocarboyxlate ligands such as EDTA or CDTA and their derivatives may form seven-coordinate complexes with a single inner-sphere water, or may form dimeric complexes at neutral or basic pH, depending on the chelate backbone and pendant groups [32]. For Fe(III) complexes that have an inner-sphere water ligand rather than an hydroxide, a disadvantage is that exchange of bound and bulk water is typically slower for Fe(III) complexes than it is for most Gd(III) or Mn(II) complexes [11,33,34]. The water exchange rate constant is typically in the range of 10^6^–10^8^ s^−1^ for Gd(III) or Mn(II) complexes, with optimal rate constants of 10^7^–10^8^ s^−1^ at moderate field strengths of 3 T [7]. In fact, the potentially complicated solution chemistry of Fe(III) may be bypassed by eliminating inner-sphere water molecules and relying on second-sphere water contributions to relaxivity [19]. Additional coordination chemistry challenges include ensuring that the iron center is stabilized in the trivalent, high-spin state, even under the reducing conditions in the body.

Potential advantages of Fe(III) complexes that may favor their application as r_1_ relaxivity agents are the short distance between the metal center and water protons, as well as the highly polarizing nature of the trivalent iron ion. The short metal-to-water proton distance will favor greater longitudinal (T_1_) relaxation with a 1/r^6^ dependence according to Solomon–Bloembergen–Morgan theory of paramagnetic relaxation [28,35]. Moreover, one might imagine that the short bond distances and polarizing nature of the Fe(III) center would also increase second-sphere water contributions through more effective interaction with the Fe(III) center. 

To address these challenges, we recently reported on a class of Fe(III) macrocyclic complexes that featured a highly stabilized high-spin Fe(III) center, a coordination site for water, and a tunable ancillary pendant. One member of this class produces r_1_ relaxivity values at 4.7 T that are similar to that of Gd(DTPA) in blood serum as well as much higher kidney specific enhancement in T_1_-weighted mice MRI studies [19]. (DTPA is diethylenetriamine pentaacetic acid and the Gd(DTPA) complex is known as Magnevist™). To our surprise, none of the four related Fe(III) complexes that we studied contained rapidly exchanging water ligands as shown by variable-temperature ^17^O-NMR studies. Nonetheless, the inner-sphere water appeared to be necessary to produce high-r_1_-relaxivity Fe(III) contrast agents, presumably through optimizing second-sphere water interactions. Minor changes in the ancillary pendants affected both the relaxivity in solution and the enhanced MRI contrast observed in mice. While changes in solution relaxivity were dominated by second-sphere water interactions, the in vivo contrast enhancement and pharmacokinetic clearance of the agents were related to charge and lipophilicity differences of the complexes [13,20,36,37,38,39].

It is challenging to predict the pharmacokinetic clearance of a small hydrophilic contrast agent in animals, especially for Fe(III) macrocyclic complexes which are so little studied to date. The most lipophilic complex in our series of Fe(III) macrocyclic agents, Fe(L3)(OH_2_), was characterized by enhanced kidney contrast that was observed for up to four hours [19]. Incorporation of a carboxylate substituent into the benzyl group produced a less lipophilic Fe(III) complex that showed lower contrast enhancement of kidneys. This complex had a similar MR signal as a function of time as Gd(DTPA) in kidneys, blood, and liver. Connecting the benzyl or carboxybenzyl through a triazole group produced Fe(III) complexes with much lower r_1_ relaxivity. These large differences in contrast enhancement with small changes in ancillary group are puzzling to understand.

The lipophilicity, charge, and binding strength of contrast agents with serum proteins generally correlates to in vivo contrast enhancement and pharmacokinetic clearance rates. Binding to blood plasma proteins alters the biodistribution and clearance of the contrast agents, in part by increasing retention of the complexes in the blood. Lipophilic and anionic complexes tend to bind more tightly to human serum albumin (HSA) [37,40,41,42,43]. Structure–activity studies show that aryl groups and anionic substitutents increase HSA binding and influence the pharmacokinetics of Gd(III) and Mn(II) contrast agents [7,20,37,44]. Moreover, interaction of contrast agents with cell membrane receptor proteins may influence the clearance pathway in animals. For example, contrast agents that are taken up by organic anionic receptors on hepatocytes clear through the hepatobiliary route and are used for liver imaging [20,22,45,46]. 

In order to further elucidate the large differences in contrast enhancement that are based solely on changing one pendant of the macrocyclic ligand, we decided to study two Fe(III) analogues with aliphatic ancillaries and to compare them with the complexes containing benzyl groups. Our goal was to obtain Fe(III) complexes with excellent solubility, low plasma protein binding, and strong r_1_ relaxivity as properties that are requisite for the development of extracellular fluid contrast agents. 

## 2. Results 

The triazacyclononane (TACN) macrocycle framework was chosen based on its propensity to bind the small Fe(III) ion. The oxygen donor pendant groups stabilize the trivalent iron oxidation state and promote the formation of a high-spin complex. The chiral hydroxypropyl groups provide rigidity to the Fe(III) complexes. For the H-L1 ligand, propane-1-sulfonate was used as the third pendant group to increase the water solubility and to decrease the overall charge of the Fe(III) complex. The H-L3 ligand, which contains a benzyl group as the third pendant, was studied previously [19,26]. The benzyl and propane-1-sulfonate as non-coordinating pendant groups of H-L1 and H-L3 ligands, respectively, were designed to form Fe(III) complexes that bind an inner-sphere water in a six-coordinate complex. These three macrocycles gave us the opportunity to compare alkyl and aryl pendant groups, as well as to modulate the charge and inner-sphere water interaction of Fe(III) complexes as MRI contrast agents. 

**Figure 1:**
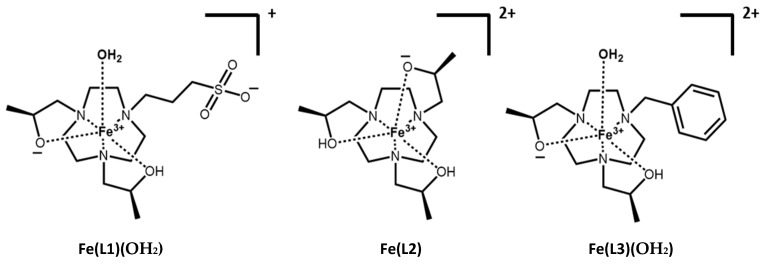
Chemical structures of the iron complexes studied here at pH 7.4. Fe(L3)(OH_2_) was synthesized and studied previously [19].

**Scheme 1:**
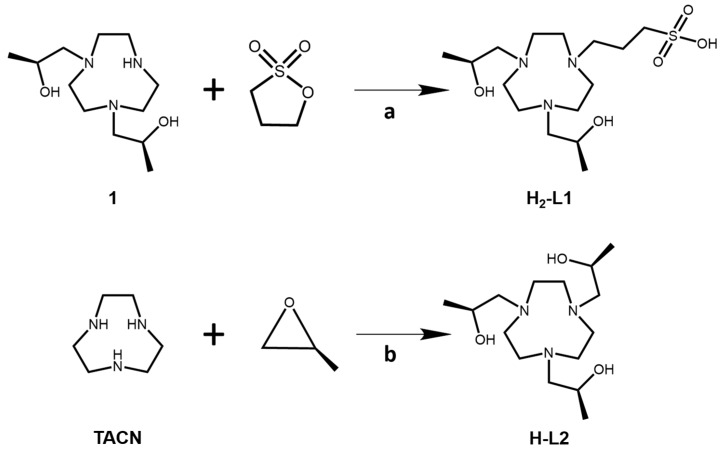
Synthesis of the ligands. (a) Acetonitrile, 1 equivalent (eq.) *N*,*N*-diisopropylethylamine and 1 eq. 1,3 propanesultone, reflux three days. (b) Ethanol, 5 eq. *S*-propylene oxide at room temperature overnight.

Key steps in the synthesis of H-L1 and H-L2 are shown in Scheme 1. Compound 1 was synthesized as previously described [19]. The H-L1 ligand was synthesized by alkylation of the secondary amine of the macrocycle by using a ring-opening reaction with 1,3 propanesultone in acetonitrile. The H-L2 ligand was prepared by alkylation of 1,4,7-triazacyclonoane (TACN) with *S*-(−)-propylene oxide in ethanol as a solvent. To prepare the Fe(III) complex, the ligand was stirred with Fe(II) chloride tetrahydrate salt in ethanol under air. The complexes were collected as yellow solids. Confirmation of the spin state of the Fe(III) complexes was obtained by using the Evans method with u_eff_ values ranging from 5.7–5.9 as shown in the Supplementary Materials (Table S1). The iron content of the complexes was determined by using ICP-MS which was consistent with an analysis of the complexes as [Fe(L1 + H^+^)Cl]Cl for Fe(L1)(OH_2_) and [Fe(L2)]Cl_2_ for Fe(L2). The ligands and their protonation states, L1, H-L1 and L2, H-L2, are defined in Figure 1 and Scheme 1.

The complexes have electronic absorbance bands at 325 nm and 330 nm with extinction coefficients ranging from 1570 M^−1^∙cm^−1^ to 3800 M^−1^∙cm^−1^ (Figures S16–S21, Table S2, Supplementary Materials). These absorbance peaks were monitored to study the dissociation of the complexes in 100 mM HCl acid solution, or in solutions containing 25 mM hydrogen carbonate and 0.40 mM hydrogen phosphate at 37 °C. In solutions containing biologically relevant concentrations of hydrogen carbonate and hydrogen phosphate, the complexes displayed no changes in their UV–Vis spectra over 72 h, consistent with no measurable dissociation of the complexes under these conditions. In acidic solution (100 mM HCl), the dissociation of Fe(L1)(OH_2_) was estimated as 18.1% over 24 h and 53.6% over 72 h based on absorbance changes. For comparison, the dissociation of the Fe(L2) complex in acidic solution (100 mM HCl) was 7.9% over 48 h, and the dissociation of Fe(L3)(H_2_O) was 14% over 24 h and 38% over 72 h [19].

The pH-potentiometric titrations of the intact Fe(III) complexes were studied to determine the equilibrium constants for deprotonation of ligand groups such as the hydroxyl pendants or water ligand. The intact complexes showed one to two ionizations over the pH range from 3 to 12 (Figures S14 and S15, Supplementary Materials). For Fe(L1)(OH_2_) and Fe(L3)(OH_2_), either an alcohol pendant group or water ligand might ionize but, for Fe(L2), we only consider alcohol pendant groups. Fe(L3)(OH_2_) has one protonated and one deprotonated hydroxy pendant at neutral pH (7.4) based on p*K*_a_ values and comparison to similar complexes [19]. For Fe(L2), one deprotonation event was observed at moderate pH values which is assigned to deprotonation of the bound hydroxyl pendant group. Fe(L1)(OH_2_) also has an ionization at close to neutral pH (7.4) which we assign to a hydroxyl group based on comparison to Fe(L2) and Fe(L3)(OH_2_). Ionization of the water ligand at near neutral pH cannot be ruled out, but is not considered to be likely based on prior studies [19]. In addition, the data for Fe(L1)(OH_2_) are best fit by including an ionization at acidic pH, although the low pH value of this ionization is superimposed on background water ionization making it difficult to determine accurately. The group that produces this ionization is not likely to be a bound water or hydroxyl group given that the coordination sphere is very similar to that of Fe(L3)(OH_2_). It is most likely that the ionization is due to the pendant sulfonic acid group in an environment that would decrease its acidity relative to typical sulfonic acid derivatives. A weak hydrogen bonding interaction to a hydroxyl pendent or bound water might produce such an environment. The complexes with their proposed protonation state at pH 7.4 are shown in Scheme 1. Speciation diagrams are shown in the Supplementary Materials (Figures S14 and S15) and calculated log K values are shown in Table 1. 

To compare the lipophilicity of the complexes, the 1-octanol/water partition coefficients (log *P*) were determined. The log P values of Fe(L1)(OH_2_) and Fe(L2) complexes are similar, but that of Fe(L3)(OH_2_) is much higher and close to 0 (Table 1), signifying a more lipophilic complex. 

**Figure 2:**
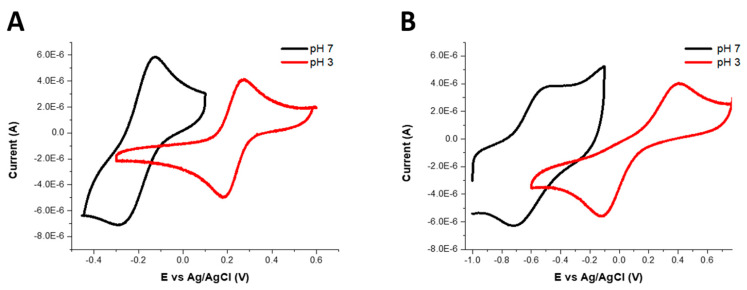
Cyclic voltammograms of 1.0 mM solutions of (**A**) Fe(L1)(OH_2_), (**B**) Fe(L2) at variable pH in water with potassium chloride (100 mM) as the supporting electrolyte and HEPES buffer. Full sweep widths were taken between −1.0 V and 1.0 V, at a scan rate of 100 mV/s for Fe(L1)(OH_2_) and 500 mV/s for Fe(L2).

Cyclic voltammetry experiments were carried out in aqueous solutions containing 0.10 M potassium chloride at acidic and neutral pH values with potassium ferricyanide salt in aqueous solution as a reference [47] (Figure S8, Supplementary Materials). The results showed that the electrode potentials of all complexes shifted to more negative values on increasing the pH from 3 to 7 (Figure 2). The electrode potential (E_1/2_) of Fe(L1)(OH_2_) at pH 3 was 227 mV vs. Ag/AgCl (515 mV vs. normal hydrogen electrode, NHE) and was −209 mV vs. Ag/AgCl (79 mV vs. NHE) at pH 7. The electrochemistry of Fe(L2) was quasi-reversible and showed two waves separated by 300 mV. At pH 3 the E_1/2_ was estimated as 264 mV vs. Ag/AgCl (552 mV vs. NHE), and, at pH 7, it was estimated as −618 mV vs. Ag/AgCl (−330 mV vs. NHE). For comparison, the E_1/2_ of Fe(L3)(OH_2_) at pH 3 was reported as 112 mV vs. Ag/AgCl (400 mV vs. NHE) and, at pH 7, the value was −588 mV vs. Ag/AgCl (−300 mV vs. NHE). 

To determine whether these complexes might be reduced to Fe(II) and react with peroxide through the Fenton process [48], the benzoate hydroxylation assay was used to study hydroxyl radical production [49]. In this test, the complexes were incubated with ascorbate as a reductant and hydrogen peroxide. Emission intensities that represent the hydroxylation of benzoate to produce salicyclic acid were compared. The Fe(III) complexes studied here did not show significant hydroxyl radical production compared to Fe(EDTA) (Figure S22 and Table S3, Supplementary Materials). This lack of reactivity suggests that the electrode potentials for all three complexes are too negative for effective redox cycling of Fe(III) to Fe(II) in the presence of the biologically relevant ascorbate ion as reductant and peroxide as oxidant [50,51,52,53].

**Figure 3:**
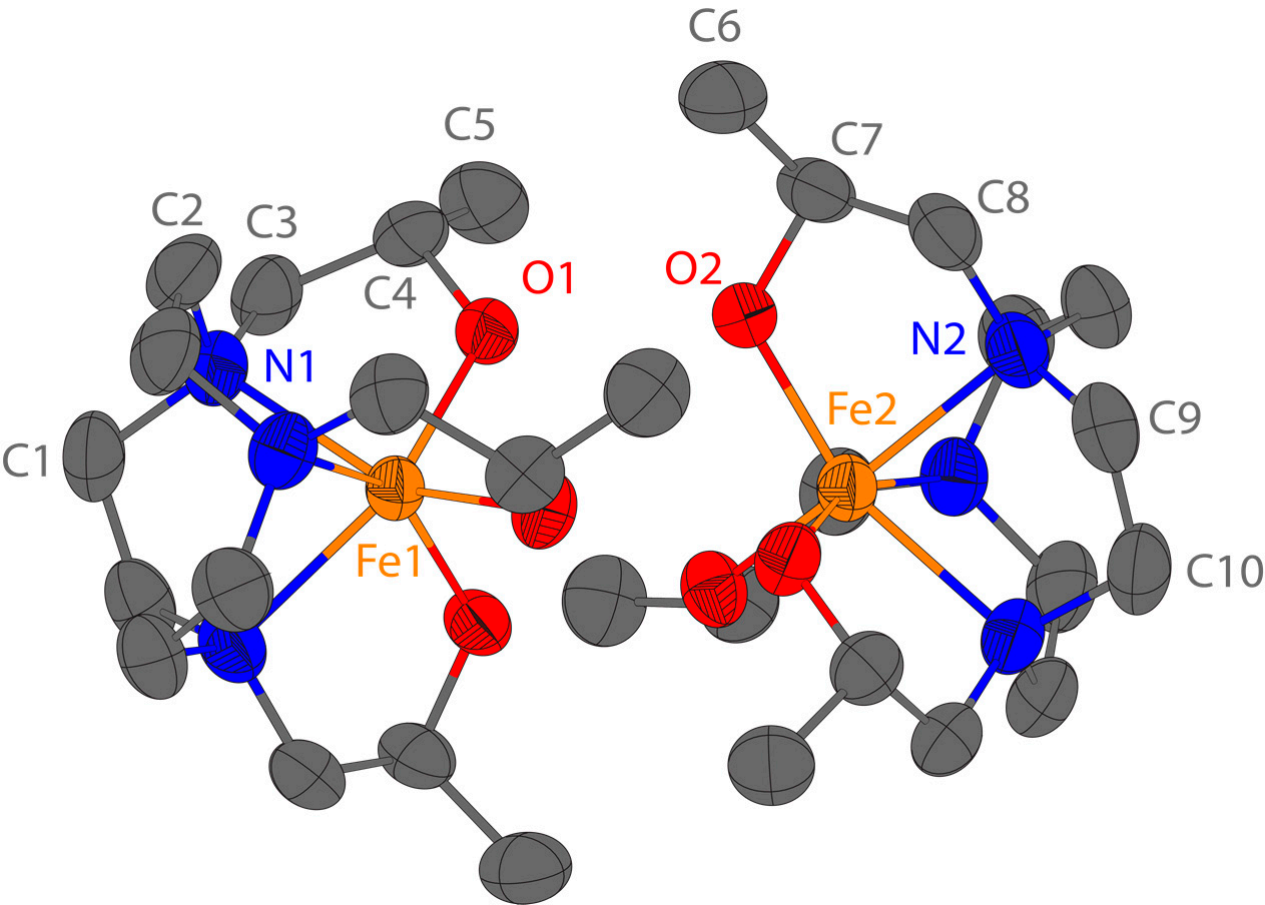
Oak Ridge Thermal Ellipsoid Plot (ORTEP) representation of [[Fe(L2)]_2_-H^+^]^3+^ extended asymmetric unit with thermal ellipsoids drawn at the 50% probability level. Hydrogen atoms were omitted for clarity.

The Fe(L2) complex crystallizes in the non-centrosymmetric space group *R*32. The asymmetric unit comprises two crystallographically unique fragments of the Fe(L2) complex and four fragments of the [FeCl_4_]^−^ anion (Figure 1). The chlorine atoms Cl1, Cl3, Cl6, and Cl8 are disordered over two positions. The two unique Fe(L2) complexes Δ(λλλ)–Fe1 and Δ(δδδ)–Fe2 are diastereomers and form dimers down the crystallographic *c*-axis. Both monomers have a six-coordinate Fe(III) center with three amine groups and three pendant alcohol groups (Figure 3). There are three [FeCl_4_]^−^ anions for every dimer, because the two anions containing iron atoms Fe3 and Fe6 have half-site occupancy. In order for the lattice to be charge balanced, the hydrogen atoms on the six pendant alcohol groups were set to half-site occupancy. The twist angles for the Fe1 and Fe2 monomers are 20.6(1)° and 39.4(1)°, respectively (Figure S23, Supplementary Materials). Similar to previously reported Fe(III) complexes of TACN with three pendants containing oxygen donors (hydroxyethyl or acetate), the Fe–N bond distances reported here are elongated from the expected value of 208 pm from the sum of the ionic radii, and the Fe–O bond distances are shorter than the expected 201 pm (Table S5, Supplementary Materials) [54,55,56]. Other trivalent metal ion complexes of L2 that are similar to the Fe(III) complex studied here include Co(III) and Cr(III) complexes. The Co(III) and Cr(III) L2 complexes also form dimers that feature hydrogen bonding between the oxygen donor atoms within the dimer [57,58].

Interactions of the two new Fe(III) complexes with water were studied by using variable-temperature ^17^O-NMR spectroscopy. Plots of the reduced ^17^O transverse relaxation rate constants as a function of inverse temperature for Fe(L1)(OH_2_) and Fe(L2) at pH 3–4 show curvature consistent with an exchanging water, similar to the classical curved plots that are fit by using Swift–Connick equations (Figures S9 and S10, Supplementary Materials) [32,59]. At more basic pH values, the appearance of the plots for Fe(L1)(OH_2_) changes, reflecting the change in speciation of the complex. The Fe(L2) complex could not be studied at pH 6–7 as it was not sufficiently soluble at the 10 mM concentrations necessary for ^17^O-NMR experiments at this pH. 

**Figure 4:**
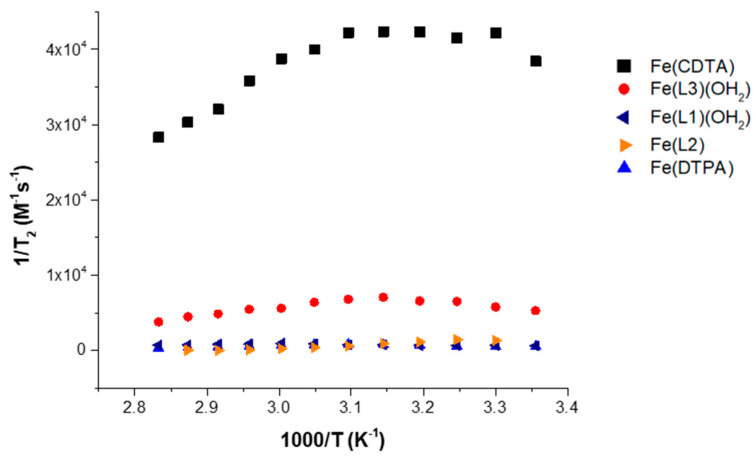
Comparison of ^17^O-NMR transverse relaxivity for Fe(L1)(H_2_O), Fe(L2), Fe(L3)(H_2_O), Fe(DTPA), and Fe(CDTA) at pH 7.4 as a function of temperature.

In comparison to reported complexes with an inner-sphere water, however, the magnitude of the transverse relaxation for the Fe(III) macrocyclic complexes is quite low (Figure 4). In this figure, Fe(DTPA) with q = 0 and Fe(CDTA) with q = 1 are shown alongside of the Fe(III) macrocyclic complexes [19,32]. Not surprisingly, Fe(L2) and Fe(DTPA) complexes that lack an inner-sphere water ligand show virtually no broadening of the ^17^O resonance of water and the corresponding transverse relaxivity is low. However, Fe(L1)(OH_2_) presumably has one bound water ligand, but also shows little resonance broadening at neutral pH (Figure S12, Supplementary Materials). The ^17^O resonance peak width of Fe(L3)(OH_2_) and Fe(L1)(OH_2_) reaches about 21% and 7%, respectively, of Fe(CDTA) at pH 3 (Figure S13, Supplementary Materials) and only about 15% and 1% of maximum peak width of Fe(CDTA) at neutral pH. This is consistent with the absence of a rapidly exchanging inner-sphere water ligand for Fe(L1)(OH_2_), similar to our observations for Fe(L3)(OH_2_) [19].

Binding of the Fe(III) complexes to human serum albumin (HSA) was studied by using ultrafiltration across a MW 5000 cutoff membrane. The solutions containing HEPES buffer (pH 7.4) and salt NaCl were incubated at 37 °C for 20 min. Solutions contained 0.4 mM Fe(III) complex and 4.5 % (*w*/*v*) HSA (0.7 mM). The binding percentages were measured by using ICP-MS to analyze the Fe concentration in the unbound complex solutions after HSA incubation (Table 1) [20,37,42,44,60]. The protein binding for Fe(L3)(OH_2_) was higher (75%) than for Fe(L1)(OH_2_) (37%) or Fe(L2) (11%).

The r_1_ and r_2_ relaxivity of the three complexes in buffer and HSA on a 4.7-T MRI scanner and 9.4-T NMR spectrometer are shown in Table 2. In buffer, the r_1_ relaxivity values of Fe(L1)(OH_2_) and Fe(L3)(OH_2_) are similar at 2.0 mM^−1^∙s^−1^ and 2.2. mM^−1^∙s^−1^, respectively, at 4.7 T. Both complexes have higher relaxivity than Fe(EDTA) (1.4 mM^−1^∙s^−1^ at 4.7 T) which has a single exchangeable inner-sphere water molecule [50]. The r_1_ relaxivity value of the Fe(L2) complex in buffer is two-fold less (1.0 mM^−1^∙s^−1^) than that of Fe(L1)(OH_2_) or Fe(L2) at 4.7 T. In HSA-containing solutions, the r_1_ relaxivity values of Fe(L1)(OH_2_), Fe(L2) and Fe(L3)(OH_2_) change slightly, consistent with weak binding to this blood protein. Gd(DTPA) relaxivity does not increase substantially in the presence of HSA. As expected, increasing the field strength from 4.7 T to 9.4 T caused a decrease in the r_1_ relaxivity value of Gd(DTPA) to 2.5 mM^−1^∙s^−1^ from 3.1 mM^−1^∙s^−1^ [19]. Notably, the increase in field strength did not substantially affect the r_1_ relaxivity values of the Fe(III) complexes. This peculiar feature of Fe(III) complexes was predicted from simulation of NMRD profiles [28].

**Figure 5:**
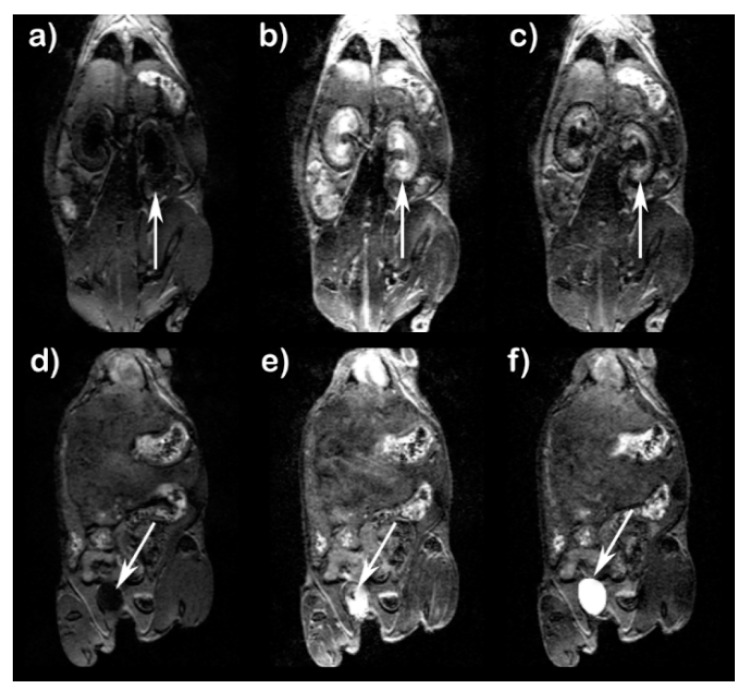
T_1_-weighted MRI of a healthy Balb/C mouse at 4.7 T at a dose of 0.20 mmol/kg Fe(L1)(OH_2_). Top row: before (**a**), after 5 min (**b**), and after 40 min (**c**) post-injection images showing enhancement of kidneys (arrow). Bottom row: urinary bladder images (arrow) before (**d**), after 5 min (**e**), and after 40 min (**f**).

The two new Fe(III) complexes, Fe(L1)(OH_2_) and Fe(L2), and the Gd(III) agent, Gd(DOTA), were studied in Balb/c mice on a small animal 4.7-T MRI scanner upon tail vein injection of the agent (Figure S24, Supplementary Materials). The excellent aqueous solubility of Fe(L1)(OH_2_), which is much higher than that of Fe(L2), allowed us to study several doses including 0.05, 0.10, and 0.20 mmol/kg that were tolerated well by the mice (Figure 5). Biodistribution and pharmacokinetic clearance of the complexes in mice were monitored by using dynamic MRI. 

**Figure 6:**
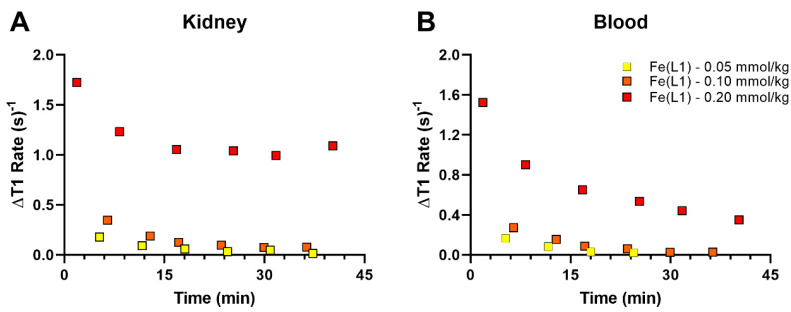
Changes in T_1_ rates for Fe(L1)(OH_2_) over time in the kidneys (**A**) and blood (vena cava) (**B**) in healthy Balb/C mice at 4.7 T at 0.05, 0.1, and 0.2 mmol/kg.

Figure 5 shows T_1_-weighted images of kidneys and urinary bladder of mice at 5 min and 40 min after treatment with Fe(L1)(OH_2_). Enhancement of kidney contrast was observed at 5 min post-injection. At 40 min post injection, images show a marked decrease in kidney contrast enhancement and an increase in urinary bladder enhancement. Unlike Fe(L3)(OH_2_), no prolonged enhancement of liver or gall bladder was observed, suggesting that Fe(L1)(OH_2_) was cleared predominately by the renal system. Pharmacokinetic data are shown in Figure 6 at three different doses of Fe(L1)(OH_2_), with the lowered doses of 0.10 and 0.05 mmol/kg, showing less blood and kidney enhancement than at 0.20 mmol/kg (Figure 6).

For comparison, changes in T_1_-weighted contrast enhancement for Fe(L1)(OH_2_), Fe(L2), and Fe(L3)(OH_2_), as well as for Gd(DOTA), over time (45 min) in the kidneys, the liver, and the blood vessel (inferior vena cava) in a healthy mouse at 0.05 mmol/kg are shown in Figure S24 (Supplementary Materials). The contrast enhancement for Fe(L3)(OH_2_) in kidneys at 15 min is much stronger than that of Fe(L1)(OH_2_) or Gd(DOTA) at 0.05 mmol/kg doses. Fe(L2) shows slightly more enhanced contrast in kidneys and blood than Fe(L1)(OH_2_) or the Gd(III) complexes at 0.05 mmol/kg from 5–30 min (Figure S24, Supplementary Materials).

## 3. Discussion

The two new Fe(III) complexes prepared and studied here, Fe(L1)(OH_2_) and Fe(L2), have an aliphatic group as the third macrocycle pendant, whereas Fe(L3)(OH_2_) and its three other analogues (Fe(L4)–Fe(L6) in Figure S1, Supplementary Materials) contained an aryl group. This simple change produced subtle differences in solution chemistry of the complexes. Fe(L1)(OH_2_) has an ionization assigned to a hydroxypropyl pendant which is slightly higher than that of Fe(L3)(OH_2_). This is consistent with the more electron-withdrawing nature of the benzyl group that produces a more Lewis acidic Fe(III) center and a more readily ionized pendant group. By comparison Fe(L2) has an ionized hydroxyl group with a pK_a_ nearly one unit lower than that of Fe(L1)(OH_2_). The lowered pK_a_ is attributed to a distinct coordination sphere that has three hydroxy pendants and no water ligand. All complexes remained largely intact in strongly acid solutions for several hours, making it feasible to carry out pH-potentiometric titrations of the complexes over a wide pH range.

The aliphatic ancillary group in Fe(L1)(OH_2_) produces an Fe(III) center which is less negative by nearly 400 mV at neutral pH in comparison to that of Fe(L3)(OH_2_), consistent with a less stabilized trivalent Fe(III) center. Nonetheless, the electrode potentials for the macrocyclic Fe(III) complexes are sufficiently negative that reduction to Fe(II) under biological conditions is unlikely [62]. Thus, incubation of Fe(L1)(OH_2_) or Fe(L2) the presence of ascorbate and peroxide does not produce hydroxylation of aromatics by Fenton chemistry, whereas complexes such as Fe(EDTA) which have very positive electrode potentials do promote the hydroxylation of aromatics under these conditions [49,50].

In our experience, it can be challenging to grow crystals of Fe(III) complexes that contain a bound water ligand, such as Fe(L1)(OH_2_). In lieu of a structure of this complex, we obtained a structure of the coordinatively saturated Fe(L2) complex. The Fe(L2) complex crystallized in a dimeric structure with the Fe(III) monomers presenting their hydroxyl face toward each other with the production of hydrogen bonds between hydroxyl groups. Each Fe(III) is six-coordinate, but with different twist angles. The twist angle of 20.6° for one monomer suggests a distorted trigonal prismatic geometry. The second monomer has a larger twist angle of 39.4° which places it closer to a distorted octahedral geometry. These differences in twist angle are consistent with the diastereomeric nature of the two complexes. At this juncture, it is not clear whether just one or both diastereomers are present in solutions of the complex as the ^1^H-NMR of the complex is severely broadened. However, Co(II) complexes with similar ligands appear to have more than one diastereomer in solution based on their ^1^H-NMR spectrum [63]. 

In aqueous solution, we propose that the Fe(L1)(OH_2_) complex has a water ligand in the sixth site. Studies on the similar Fe(L3)(OH_2_) complex suggested that the chloride anion, originally bound to the Fe(III) center, was displaced by water in aqueous solutions [19]. The limited broadening of the ^17^O resonance in variable-temperature NMR studies suggests that this bound water is not in rapid exchange with bulk water. The lack of ^17^O resonance broadening for Fe(L1)(OH2) resembles that of Fe(III) complexes with hexadentate polyaminocarboxylate ligands that form six-coordinate complexes with no inner-sphere water [32], whereas the seven-coordinate Fe(CDTA) complex has a rapidly exchanging inner-sphere water and produces substantial ^17^O resonance broadening. We attribute the slow rate of exchange of the water ligand in Fe(L1)(OH_2_) to the shorter and stronger Fe–O bonds in the six-coordinate complex compared to a seven-coordinate complex such as Fe(EDTA)(OH_2_). The lack of water exchange for Fe(L1)(OH_2_) and Fe(L3)(OH_2_) suggests that water proton relaxivity is mediated through strong second-sphere water interactions.

Curiously, the “static” water ligands in Fe(L1)(OH_2_) and Fe(L3)(OH_2_) produce complexes with higher relativity than the Fe(L2) complex which lacks an inner-sphere water. The higher relaxivity is promoted by strong second-sphere water interactions that are mediated by the water ligand in conjunction with the hydroxypropyl pendant groups. Notably, both Fe(L1)(OH_2_) and Fe(L3)(OH_2_) have higher relaxivity than Fe(EDTA) (1.4 mM^−1^∙s^−1^ at 4.7 T) which has an exchangeable water ligand [18]. Similarly, the relaxivity of Fe(L2) is higher than that of Fe(DTPA) at 0.51 mM^−1^∙s^−1^, with both complexes lacking a bound exchangeable water [18]. These comparisons suggest that the hydroxy groups of the macrocyclic complexes, as well as the static water ligand, serve to enhance second-sphere water interactions.

Interestingly, the Fe(III) complexes studied here either showed little change or an increase in r_1_ relaxivity upon increasing the field strength from 4.7 to 9.4 T (Table 2), in contrast to Gd(III) complexes [1]. This is consistent with predictions from theoretical calculations that Fe(III) complexes would show improved relaxivity at magnetic field strengths greater than 100 MHz [28]. Studies on Fe(III)-loaded synthetic melanin nanoparticles showed an increase in r_1_ relaxivity between 40 and 100 MHz [63]. This improved relaxivity at high field strength is attributed to field-dependent effects on electronic relaxation times of the Fe(III), with the electronic relaxation time being a limiting factor at low field strengths [28].

Fe(L1)(OH_2_) produces enhanced bladder and kidney contrast with no detectable gall bladder enhancement, consistent with clearance through a renal pathway. Higher doses (0.20 mmol/kg) of Fe(L1)(OH_2_) show higher kidney contrast enhancement and more pronounced MRI signal enhancement in blood. By comparison, Fe(L2) shows enhanced contrast in kidney, vena cava, and liver at 15 min which is slightly higher than that of Gd(DTPA) or Gd(DOTA) at the same dose. Unfortunately, Fe(L2) could not be studied at typical doses of 0.10 or 0.20 mmol/kg due to its moderate solubility. 

What then contributes to the differences in enhanced contrast that are observed in mice? In particular, Fe(L3)(OH_2_) shows much higher contrast at 10–15 min than all of the other complexes at doses of 0.05 mmol/kg in kidneys and vena cava. This may be due, in part, to tighter binding of the more lipophilic Fe(L3)(OH_2_) complex to blood proteins such as serum albumin in comparison to other complexes studied here. Binding to serum albumin is anticipated to promote retention in the bloodstream. Also of note is the unusual coordination chemistry of the dicationic yet lipophilic Fe(L3)(OH_2_) complex. We recently showed that Fe(L3)(OH_2_) binds to yeast glucan nanoparticles and to ligands with anionic oxygen donors [26]. The coordination chemistry of this cationic complex combined with its relatively lipophilic nature may be the source of the prolonged retention in kidneys that is lacking for Fe(L1)(OH_2_) or Fe(L2). Interactions of lipophilic cationic dyes with receptors on the kidney cell surface such as organic cation transporters were reported [64,65]. Further studies on Fe(III) complex solution chemistry and the role of the ancillary pendant in modulating binding to biomacromolecules are warranted to better understand these differences.

## 4. Materials and Methods

### 4.1. Materials 

*N*,*N*-Dimethylformamide dimethyl acetal (97%), benzyl bromide (99%), palladium 10% on carbon (Type 487) and l-ascorbic, HEPES (99%), and sodium chloride (99%) were purchased from Alfa Aesar (Haverhill, MA, USA). 1,4,7-Triazacyclononane (97%), 1,3 propanesultone and (*S*)-(−)-propylene oxide (>98%) were purchased from TCI America (Portland, OR, USA). Ferrous chloride tetrahydrate (99%) was purchased from Strem Chemicals (Newburyport, MA, USA). Column chromatography was performed using alumina gel, basic Brockman Activity 1 60 × 325 mesh from Fisher Scientific (Hampton, NH, USA). Tetrahydrofuran was dried over molecular sieves, grade 514 type 4A; 8–12 mesh beads that were purchased from Fischer Scientific (Hampton, NH, USA). Albumin from human serum (fatty acid free) was purchased from Sigma-Aldrich (St. Louis, MO, USA). *N*,*N*-Diisopropylethylamine (DIEA) was purchased from Beantown Chemical (Hudson, NH, USA). 

### 4.2. Instrumentation

A Varian Inova 500-MHz NMR spectrometer equipped with FTS Systems TC-84 Kinetics Air Jet Temperature Controller was used to collect ^1^H-NMR spectra (Varian, Palo Alto, CA, USA). A Varian Mercury 300-MHz NMR spectrometer (Palo Alto, CA, USA) operating at 75 MHz was used to collect ^13^C-NMR spectra. Varian Inova 400-MHz spectrometer (Palo Alto, CA, USA) equipped with a 5-mm broad-band probe operating at a resonance frequency of 54.24 MHz were used for ^17^O-NMR experiments. All pH measurements were performed by using an Orion 8115BNUWP Ross Ultra Semi Micro pH electrode connected to a 702 SM Titrino pH. A Thermo Finnigan LCQ Advantage (Thermofisher, Waltham, MA, USA) with ESI ionization and Surveyor HPLC and a 12T Bruker SolariXR 12 Hybrid FTMS (Bruker Scientific, Billerica, MA, USA) with Imaging MALDI and Nano-LC were used for analyzing masses of the complexes and the ligands. Iron concentration was determined using a Thermo X-Series 2 ICP-MS (Thermofisher, Waltham, MA, USA). Absorbance spectra were recorded on a Beckman-Coulter DU 800 UV–Vis Spectrophotometer (Beckman Coulter, Pasadena, CA, USA) equipped with a Peltier temperature controller. VersaSTAT 3 potentiostat from Princeton Applied Research was used for cyclic voltammograms that were obtained by using a glassy carbon working electrode (CH Instruments Inc.), Ag/AgCl reference electrode (CH Instruments Inc., Austin, TX, USA), and platinum wire auxiliary electrode. A Bruker SMART APEX II CCD diffractometer (Bruker, Billerica, MA, USA) was used for crystallographic experiments. 

### 4.3. Synthesis

*3-(4,7-bis((S)-2-hydroxypropyl)-1,4,7-triazonan-1-yl)propane-1-sulfonate,***H_2_-L1**. Compound **1** (Scheme 1 (0.100 g, 0.407 mmol) that was synthesized according to the synthesis procedure described in Reference [19] was dissolved in 15.0 mL of acetonitrile in the 25-mL round-bottom flask. Two equivalents of 1, 3-propanesultone (0.099 g, 0.813 mmol) and one equivalent of DIEA were added and brought to reflux for three days. The solvent was removed on a rotoevaporator and the residue was dried on a Schlenk line under vacuum. **H_2_-L1** ligand (Scheme 1) was purified on a basic alumina column by eluting with dichloromethane and increasing the volume percentage of methanol. **H_2_-L1** eluted at 4%–8% by volume methanol. ^1^H-NMR, 500 MHz (D_2_O, ppm) δ: 3.99 (s, –CH–, 2H); 2.62–3.01 (m, –CH_2_–, 20H); 1.92 (q, –CH_2_–, 2H); 1.10 (d, –CH_3_, 6H) ^13^C-NMR (75 MHz, D_2_O) δ: 19.9, 21.3, 47.3, 48.5, 50.4, 54.1 (Figures S2 and S3, Supplementary Materials) M: H_2_-L1; ESI-MS (0.1% formic acid method), found *m/z* 368.3 (M + H^+^, 100 %) and *m/z* 390.3 (M + Na^+^, 27%). The compound was isolated as white-beige solid (55.5 mg, 37%).

*(2S,2′S,2″S)-1,1′,1″-(1,4,7-triazonane-1,4,7-triyl)tris(propan-2-ol),***H-L2**. To a 25-mL round-bottom flask with gas inlet and stir bar was added TACN (0.100 g, 0.774 mmol) and five equivalents of *S*-propylene oxide (0.225 g, 3.87 mmol.). The solution was stirred for one day under Ar at room temperature. The solvent was removed under vacuum and the residue was dried on a Schlenk line under vacuum. ^1^H-NMR, 400 MHz (CDCl_3_, ppm): 3.84 (s, 3H, OH); 2.84 (m, 3H, CH); 2.97 (m, 6H, pendant CH_2_); 2.40 (m, 4H, ring CH_2_); 2.65 (m, 12 H, ring CH_2_); 1.09 (d, 12H, CH_3_). ^13^C-NMR in CDCl_3_: δ 66.4, 63.4, 52.7, 19.8 [66,67]. M: H-L2; ESI-MS, found *m/z* 304.4 (M + H^+^, 100%), *m/z* 326.4 (M + Na^+^, 25 %) and *m/z* 102.1 ((M + 3H^+^)/3, 10%). The compound was isolated as white-beige solid (0.229 g, 97%).

**[Fe(L1+H^+^)Cl]Cl.** The **H-L1** ligand (0.100 mmol) was dissolved in ethanol (5.00 mL)**.** Then, ferrous chloride tetrahydrate (1 equivalent, 0.100 mmol) was dissolved in ethanol (2.00 mL) and was added dropwise to the ligand solution. Fe(L1) complex was isolated as yellow solid (26.1 mg, 73%) by precipitation with diethyl ether. Fe content was calculated through ICP-MS for [Fe(L1 + H^+^)Cl]Cl: 11.38%, found: 11.57% ± 0.01%. M: [Fe(L1)]^+^; LCQ-MS: found *m/z* 421.2 (M, 70%), *m/z* 443.2 (M + Na^+^ − H^+^), 30%), *m/z* 841.0 (2M − H^+^, 100%), and *m/z* 863.0 (2M + Na^+^ − 2H^+^, 18%). FT-ICR-MS: calculated *m/z* 421.13341, found *m/z* 421.13273 (M, 100%), calculated *m/z* 443.11535, found 443.11460 (M + Na^+^ − H^+^, 43%). 

**[Fe(L2)]Cl_2_**. The **H-L2** ligand (0.100 mmol) was dissolved in ethanol (5.00 mL)**.** Then, ferrous chloride tetrahydrate (1 equivalent, 0.100 mmol) was dissolved in ethanol (2.00 mL) and was added dropwise to the ligand solution. Fe(L2) complex was precipitated as yellow solid (21.5 mg, 51%). The yellow solid complex was washed with diethyl ether. Fe content was calculated through ICP-MS for [Fe(L2)]Cl_2_: 13.08%, found: 12.85% ± 0.09%. M: [Fe(L2)]^2+^; LCQ-MS: found *m/z* 357.2 (M − H^+^, 100%), *m/z* 713.1 (2M − 3H^+^, 40%), and 735.1 (2M + Na^+^-4H^+^, 17%). FT-ICR-MS: calculated *m/z* 357.17150, found *m/z* 357.17820 (M − H^+^, 100%), calculated *m/z* 713.33517, found *m/z* 713.33414 (2M − 3H^+^, 6.8%), calculated *m/z* 735.31711, found *m/z* 735.31576 (2M + Na^+^ − 4H^+^, 1.5%).

### 4.4. ^17^O Variable-Temperature NMR Spectroscopy 

Samples were prepared in 1% H_2_^17^O water solution and studied at variable temperatures (20–80 °C). 1/T_2_ was calculated by subtracting the full width at half maximum (FWHM) of the ^17^O resonance with Fe(III) complex from that in the absence of complex [68]. The reduced transverse relaxation times of ^17^O-NMR resonances were calculated and fit by using the Swift–Connick equation [66,69].

### 4.5. Magnetic Susceptibility

The effective magnetic moment (µ_eff_) was calculated by using Evans method [70,71] (Equations (1)–(3)). Samples were prepared by using a coaxial NMR insert with an outer 5-mm NMR tube. The NMR insert contained a solution of 5% *t*-butanol and 95% D_2_O by volume and 4.00 mM complex. The outer NMR tube contained 5% *t*-butanol and 95% D_2_O by volume. Experiments were carried out at 298 K (T). Measurements were made in triplicate, and the averaged values are presented in Table S1 (Supplementary Materials).

(1) $$\chi_{g}=\frac{-3\Delta f}{4\pi v_{0}m}+\chi_{0}+\frac{\left[\chi_{0}\left(d_{0}-d_{s}\right)\right]}{m},$$

(2) $${µ}_{eff}=2.83{\left(\chi_{m}T\right)}^{1/2}$$

The mass susceptibility ($\chi_{g}$) was calculated using Equation (1), where Δf is the shift in frequency (Hz), ν_0_ is the operating frequency of NMR spectrometer (Hz), *m* is the concentration of the substance (g/mL), *do* and *d_s_* are the densities of pure solvent and solution, and $\chi_{0}$ is the mass susceptibility of the solvent ($\chi_{0}$ = −0.6466 × 10^−6^ cm^3^/g) [72]. The molar susceptibility ($\chi_{m}$) is obtained by multiplying the mass susceptibility ($\chi_{g}$) by the molar mass. This result was used to calculate the effective magnetic moment *μ_eff_* (Equation (2)).

### 4.6. Oxidation of Benzoate Assay

Production of oxidizing species by the Fe(III) complexes was tested by using benzoate hydroxylation fluorescence assay [49]. The samples were prepared with 1.00 mM benzoate, 50.0 μM Fe(III) complex, 50.0 μM ascorbate as a reducing agent, 50.0 μM H_2_O_2_, 100.0 mM NaCl, and HEPES buffer (pH 7.4, 20.0 mM). The samples were incubated at 37 °C for 1h. Control sample was prepared with all reagents except Fe(III) complexes. Both samples and control were excited at 308 nm, and fluorescence was observed at 410 nm from the salicylic acid product.

### 4.7. ICP-MS Measurement 

A Thermo X-Series 2 ICP-MS was used to determine Fe concentration of the Fe(III) complexes. The samples were diluted from 10.0 mM to 1.00 mM Fe concentration solutions. Then, 100.0-µL (1.00 mM) sample solutions were dissolved in 90% *v/v* metal-free HNO_3_ (total 1.00 mL) for digestion. After a three-day digestion process, they were diluted to 2% HNO_3_ and 50 ppb internal solution in 10.0 mL of Milli-Q water. A linear calibration curve for iron metal ranging from 0.1 ppb to 100 ppb was prepared for the quantification. As an internal standard, cobalt standard solution was used. Data analysis was performed by using PlasmaLab software.

### 4.8. Electrochemistry Experiments

Cyclic voltammetry measurements were performed in 10.0-mL aqueous solutions containing 0.100 M potassium nitrate as supporting electrolyte and 1.00 mM Fe(III) complexes. A standard three-electrode cell was used with a polished glassy carbon as the working electrode, a Pt wire as the auxiliary electrode, and an Ag/AgCl (1.00 mM) reference electrode. The samples were purged with extra pure N_2_ gas to remove the dissolved oxygen for each measurement. Current was measured with a 10-s prescan delay between −1.5 V and 1.5 V at a sweep rate of 100 and 500 mV/s, where 1.00 mM ferrocyanide was used as a standard solution. The redox potential of Fe(II)/(III) was calculated from the anodic (E_pa_) and cathodic (E_pc_) potentials, as E_Fe(II)/(III)_ = (E_pa_ + E_pc_)/2 in reference to Ag/AgCl (1.00 mM), then converted to a value referenced to the normal hydrogen electrode (NHE). 

### 4.9. Log P Measurements 

Fe(III) complex solutions (0.500 mM) were prepared in 1.00 mL of H_2_O with 20.0 mM HEPES buffer (pH 7.4) and 100.0 mM NaCl. 0.500 mL of Fe(III) complex solution and 0.500 mL of 1-octanol was prepared. The mixture was shaken for 24 h, then centrifuged at 13,000 rpm for 1 min. Then, the solution was allowed to stand for 1 h. Iron concentration in the aqueous layer before and after mixing with 1-octanol was determined by ICP-MS. The partition coefficient was calculated from Equation (3) where Co is the concentration of iron in the 1-octanol layer and Cw is the concentration of iron in the water layer [39].

(3) $$logP=\log\left(\frac{Co}{Cw}\right).$$

### 4.10. UV–Vis Spectroscopy

Absorbance of Fe(III) complexes was measured from 200–800 nm over a period of 48 and 72 h. A Peltier temperature controller was used to keep the temperature at 37 °C. The sample solutions contained 0.200 mM Fe(III) complex. Solutions for kinetic inertness experiments contained 25.0 mM NaHCO_3_, 0.500 mM Na_2_HPO_4_, and 10.0 mM HEPES buffer (pH 7.4). Acidic solutions for the kinetic inertness experiments contained 0.10 M HCl. For each of these experiments, extinction coefficients were determined by using by using the Beer–Lambert law as given in Table S2 (Supplementary Materials).

### 4.11. Binding Measurements of Fe(III) Complexes to Human Serum Albumin (HSA)

The molecular weight of 66,435 Da was used to determine the molar concentrations of HSA. Fe(III) complex (0.400 mM) solutions were incubated with 4.5% (*w*/*v*) HSA in 0.100 M NaCl and 20.0 mM HEPES buffer (pH 7.4). All solutions were incubated at 37 °C for 20 min [42]. Aliquots (0.300 mL) were placed in ultra-centrifugal filter units with a 5-kDa membrane and centrifuged at 3500× *g* for 10 min. The filtrates were used to determine Fe concentration from digested unbound Fe(III) complexes by using ICP-MS. 

### 4.12. pH Potentiometric Titrations

All solutions were prepared by using carbonate-free water. 

Fe(L1)(OH_2_): The solution contained 0.527 mM Fe(III) complex added as the chloride salt, 1.00 mM meglumine, 0.100 M NaCl, and 3.72 mM HCl to decrease the pH of the solution to pH 3 in total volume of 20.00 mL. The solution was titrated with a 98.83 mM NaOH solution. Then, 5.00 µL aliquots of the NaOH solution were added over 60 s with an interval of 300 s using a Nexus 3000 High Flow Syringe Pump from Chemyx Inc (Stafford, TX, USA) equipped with a 10-mL Hamilton gastight syringe. 

Fe(L2): The solution contained 0.75 mM Fe(III) complex added as the chloride salt, 2.00 mM meglumine, 0.100 M NaCl, and 6.00 mM HCl to decrease the pH of the solution to pH 3 in total volume of 25.00 mL. The solution was titrated with a 94.86 mM NaOH solution. Then, 5.00 µL aliquots of the NaOH solution were added over 60 s with an interval of 300 s using a Nexus 3000 High Flow Syringe Pump from Chemyx Inc (Stafford, TX) equipped with a 10-mL Hamilton gastight syringe. 

In all fits of the data, meglumime was used as a third reagent, and the equilibrium equation corresponding to its ionization was added to the model data fit as determined from separate titrations. The titration was completed under Ar(g) at 25 °C. The titrations were monitored from pH 3 to pH 11. Equilibrium constants for all complexes were determined by fitting the data using HYPERQUAD 2013 Version 6.0.1. Speciation diagrams were plotted by utilizing HySS Version 4.0.31.

### 4.13. T_1_/T_2_ Relaxivity Measurements

Relaxivity of the complexes was determined at 4.7 T (small animal MRI) and 9.4 T (400 MHz NMR). Both T_1_ and T_2_ experiments were performed at 37 °C for the concentration range of 50.0 µM to 0.400 mM Fe(III) complex. The solutions contained 0.100 M NaCl and HEPES buffer (pH 7.4, 20 mM). An inversion recovery True FISP acquisition was used to measure T_1_ relaxation rate constants. T_2_ relaxation rates were measured by using multi-echo, Carr–Purcell–Meiboom–Gill spin-echo sequence with a fixed TR of 3000 ms and TE times ranging from 20–1200 ms. The T_1_ and T_2_ relaxivity values were calculated by using linear regression fitting of 1/T_1_ (s^−1^) and 1/T_2_ (s^−1^) versus concentration (mM) in Microsoft Excel.

### 4.14. Mice Imaging

In vivo imaging studies with the Fe(III) complexes were studied on at 4.7 T Bruker preclinical MRI in mice (BABC/cJ, Jackson Laboratory) in accordance with approved Institutional Animal Care and Use Committee (IACUC) protocols. The samples with a concentration of 0.050, 0.100, and 0.200 mmol [Fe]/kg. The Fe(III) complex stock solutions were adjusted to pH 7.2 by addition of meglumine and were injected intravenously via tail vein. MR data were recorded continuously for up to 1 h. Distribution studies and clearance kinetics were studied by using MR data acquired over several time points for up to 1 h, and additionally at 4 h and 24 h. For comparison, The Food and Drug Administration (FDA)-approved MRI contrast agent gadopentetate dimeglumine (Gd(DTPA), Magnevist®) and gadoterate meglumine (Gd(DOTA), Dotarem®) were injected at 50.0 μmol [Gd]/kg into separate group of mice and imaged as above. 

### 4.15. X-ray Structure Solution and Refinement

Single crystals of [Fe(L2)]_2_-H^+^][FeCl_4_]_3_ were grown through slow evaporation of solvent from a water/methanol mixture. Data were collected on a Bruker SMART APEX II CCD diffractometer. Data reduction was completed using SAINT version 8.40A, and a multi-scan absorption correction was applied using SADABS version 2016 included in the Bruker APEX3 software suite [73]. Space-group determination was performed using the XPREP utility included in the SHELXTL software package [74]. The structure was solved with ShelXT [75] using intrinsic phasing and refined with ShelXL [76] using least squares minimization (full-matrix least-squares on F^2^). Hydrogen atoms attached to oxygen atoms were located in the difference Fourier synthesis and had their distances restrained using the DFIX command to an effective O–H distance of 0.84 Å retrieved from the lst file. Anomalous scattering was used for absolute structure determination. The twist angles were calculated as torsion angles using the Olex2 software package [77]. Crystallographic data and data collection parameters are given in Table S4 (Supplementary Materials).

## 5. Conclusions

As shown here, Fe(III) macrocyclic complexes are promising for further development as MRI contrast agents. One of the macrocyclic Fe(III) complexes studied here demonstrates promising r_1_ relaxivity in phantoms at 4.7 T that we attribute to second-sphere water interactions, similar to an analogous complex with a benzyl ancillary group. The addition of an alkyl group with anionic sulfonate has the advantage that it produces a hydrophilic Fe(III) complex of high solubility. However, the replacement of a benzyl group with an alkyl group on the TACN macrocycle produces an Fe(III) complex that is less highly stabilized in the trivalent state. The hydrophilic nature of the Fe(L1)(OH_2_) complex combined with its relatively low binding to serum albumin leads to a complex that behaves as an extracellular fluid contrast agent. In fact, Fe(L1)(OH_2_) shows a pharmacokinetic profile that is similar to that of Gd(DTPA) or Gd(DOTA). Future research will focus on the molecular properties of Fe(III) macrocyclic complexes that contribute to serum protein binding and will explore factors that modulate biodistribution of the contrast agents.

## Figures and Tables

**Table 1:** Equilibrium constants for ionization of Fe(L1)(OH_2_), Fe(L2), and Fe(L3)(OH_2_), octanol water partition coefficients and serum albumin binding.

Complex	Log *K_1_* (p*K_a2_*)^a^	Log *K_2_* (p*K_a1_*) ^a^	Log *P* ^b^	HSA Binding (%) ^c^
Fe(L1)(OH_2_)	7.55 ± 0.08	3.39 ± 0.17	−1.67	36.8 ± 0.2
Fe(L2)	11.78 ± 0.11	6.57 ± 0.05	−1.60 ± 0.1	11.2 ± 2.8
Fe(L3)(OH_2_)	7.17 ± 0.01	-	−0.13	75.1 ± 3.7

**a.** potentiometric titrations at 25 °C, in 0.10 M NaCl, with 1–2 mM meglumine. See Supplementary Materials for equilibrium expressions. **b.** Log P octanol/water partition coefficients were calculated from Equation (3). Values were compared to Fe(L3)(OH_2_)[19] **c.** HSA (0.7 mM) 4.5% (*w*/*v*) binding at 0.4 mM complex, 20 mM 4-(2-hydroxyethyl)-1-piperazineethanesulfonic acid (HEPES) buffer (pH 7.4), and 100 mM NaCl.

**Table 2:** Relaxivity values for Fe(III) complexes measured at 4.7 T and 9.4 T, pH 7.4 and 37 °C compared to Gd(III) complexes. HSA—human serum albumin.

Complex	r_1_ (mM^−1^∙s^−1^) 4.7 T	r_1_ (mM^−1^∙s^−1^) 9.4 T	r_2_ (mM^−1^∙s^−1^) 4.7 T	r_1_ (mM^−1^∙s^−1^) in HSA 4.7 T	r_2_ (mM^−1^∙s^−1^) in HSA 4.7 T
Fe(L1)(OH_2_)	2.0 ± 0.19	2.0 ± 0.2	6.1 ± 0.77	2.5 ± 0.63	5.5 ± 0.98
Fe(L2)	0.97 ± 0.12	1.4 ± 0.08	1.8 ± 0.47	1.2 ± 0.22	2.3 ± 1.1
Fe(L3)(OH_2_) ^a^	2.2 ± 0.30	------	4.5 ± 1.1	2.5 ± 0.10	4.2 ± 0.70
Gd(DTPA) ^a^	3.1	2.5	3.9	3.2	4.0
Gd(DOTA) ^b^	2.8 ± 0.10	------	3.7 ± 0.20	------	------

a. From Reference [19]; b. from Reference [61].

## Supplementary Materials

The following are available online, Chemical structures of the iron complexes; Figure S1. ^1^H-NMR spectrum of H-L1; Figure S2. ^13^C-NMR spectrum of H-L1; Figure S3. LCQ-MS spectrum of [Fe(L1+H+)Cl]Cl; Figure S4. LCQ-MS spectrum of [Fe(L2)]Cl_2_; Figure S5. Effective magnetic moment values through Evan’s method for iron complexes; Table S1. Evans method NMR spectrum of Fe(L1)(OH_2_); Figure S6. Evans method NMR spectrum of Fe(L2); Figure S7. Cyclic voltammograms of potassium ferrocyanide; Figure S8. Transverse ^17^O-NMR relaxivity, ln(1/T_2r_) as a function of temperature for Fe(L1)(OH_2_); Figure S9. Transverse ^17^O-NMR relaxivity, ln(1/T_2r_) as a function of temperature for Fe(L2); Figure S10. Shift and line broadening graphs of the ^17^O resonance of 1% H_2_^17^O standard solution; Figure S11. Shift and line broadening graphs of the ^17^O resonance of Fe(L1)(OH_2_)solutions; Figure S12. Comparison of Fe(L1)(OH_2_), Fe(L3)(OH_2_) and Fe(CDTA) 17O-NMR resonance broadening as a function of temperature; Figure S13. Speciation diagram from pH potentiometric data of Fe(L1)(OH_2_) solution; Figure S14. Speciation diagram from pH potentiometric data of Fe(L2) solution; Figure S15. UV–Vis absorbance spectra of Fe(L1)(OH_2_); Figures S16–S18. UV–Vis absorbance spectra of Fe(L2) were obtained over 24 h at 37 °C; Figures S19–S21. The molar extinction coefficient (ε) values of the Fe complexes; Table S2. Percent benzoate oxidation by Fe(III) complexes normalized to amount produced by Fe(EDTA) complex. Figure S22. Percent benzoate oxidation values by Fe(III); Table S3. ORTEP of the two Fe(L2) monomers; Figure S23. Crystal data and structure refinement for Fe(L2); Table S4. Selected bond lengths for Fe(L2); Table S5. Selected bond angles for Fe(L2); Table S6. Changes in T_1_ rates for Fe(L1), Fe(L2), Fe(L3), Gd(DTPA), and Gd(DOTA) over time; Figure S24.
