# Supplementary material for: Modulating the Properties of Fe(III) Macrocyclic MRI Contrast Agents by Appending Sulfonate or Hydroxyl Groups
Source: Molecules. 2020 May 13;25(10):2291. doi: 10.3390/molecules25102291 (PMC7288058; doi:10.3390/molecules25102291)
Supplement: Supplementary file 1 [file molecules-25-02291-s001.pdf]

Supplementary Materials

Modulating the properties of Fe(III) macrocyclic MRI contrast agents by appending sulfonate or hydroxyl groups

Didar Asik [1], Rachel Smolinski [1], Samira M. Abozeid [1], Travis B. Mitchell[1], Steven G. Turowski[2], Joseph A. Spernyak[2] and Janet R. Morrow[1,*]

[1] Affiliation 1; Department of Chemistry, University at Buffalo, State University of New York, Amherst, New York 14260, United States
[2] Affiliation 2; Department of Cell Stress Biology, Roswell Park Comprehensive Cancer Center, New York 14263 United States
* Correspondence: jmorrow@buffalo.edu

Received: date; Accepted: date; Published: date

Contents	Page
Table of Contents	S1
Synthetic Procedures and Characterization	S2-S4
Effective Magnetic Moment	S4-S5
Cyclic Voltammetry	S5
Plot of the reduced ^{17}O transverse relation rate constant vs. temperature	S6
Transverse ^{17}O NMR Relaxivity plots	S7
^{17}O NMR Resonance Broadening	S8
pH Potentiometric Titrations	S9
UV-Vis Spectra – Kinetic Stability	S10-S15
Percent Benzoate Oxidation	S16
X-Ray Crystallographic Data	S17-S19
In Vivo Imaging	S19
Pharmacokinetic Data	S19
References	S20

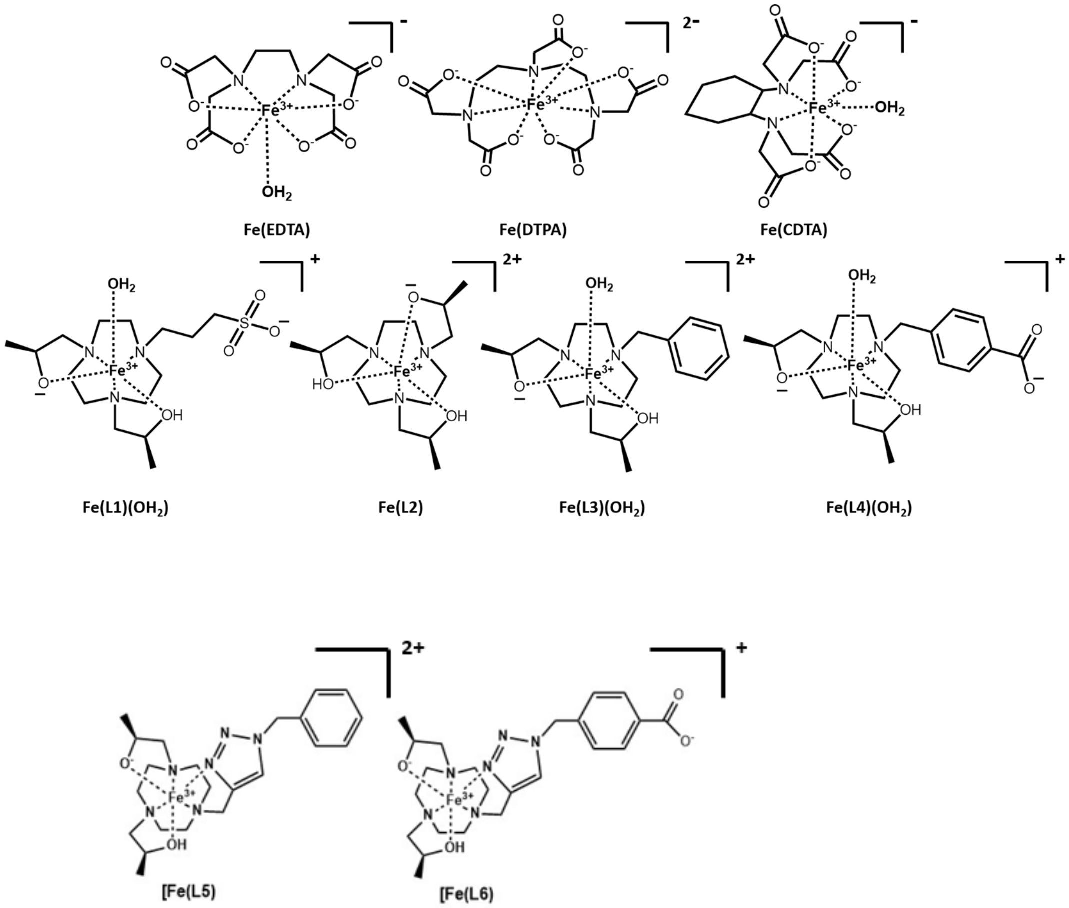

Figure S1. Chemical structures of the iron complexes studied or referenced here at pH 7.4.

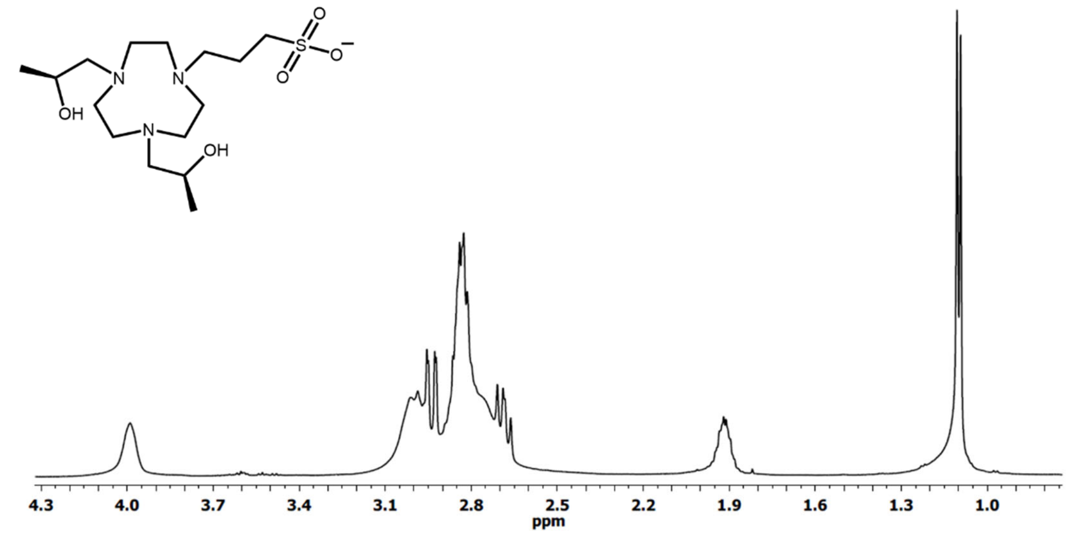

Figure S2. ^{1}H NMR spectrum of H-L1 (500 MHz, D_2O, 298 K).

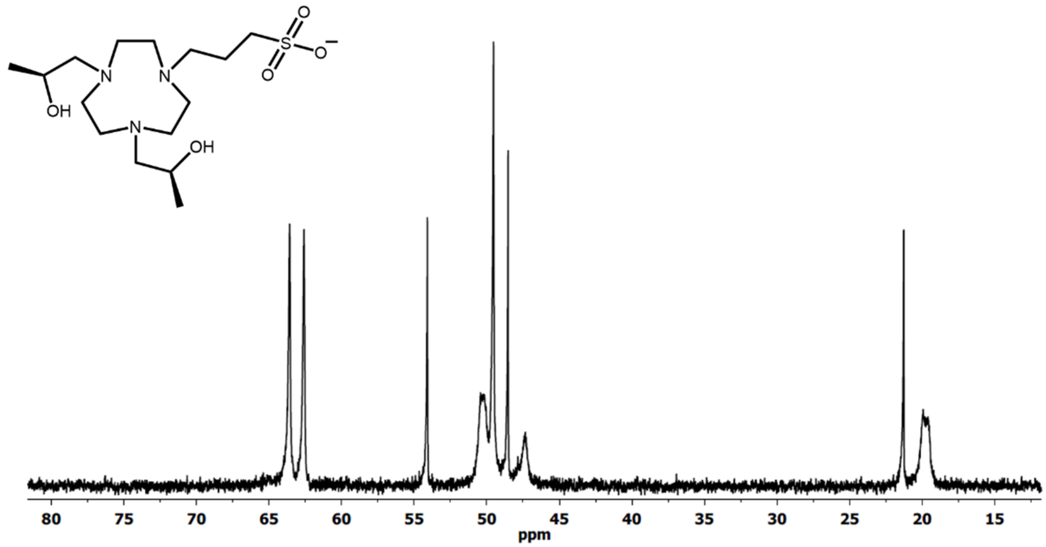

Figure S3. ^{13}C NMR spectrum of H-L1 (75 MHz, D_2O, 298 K).

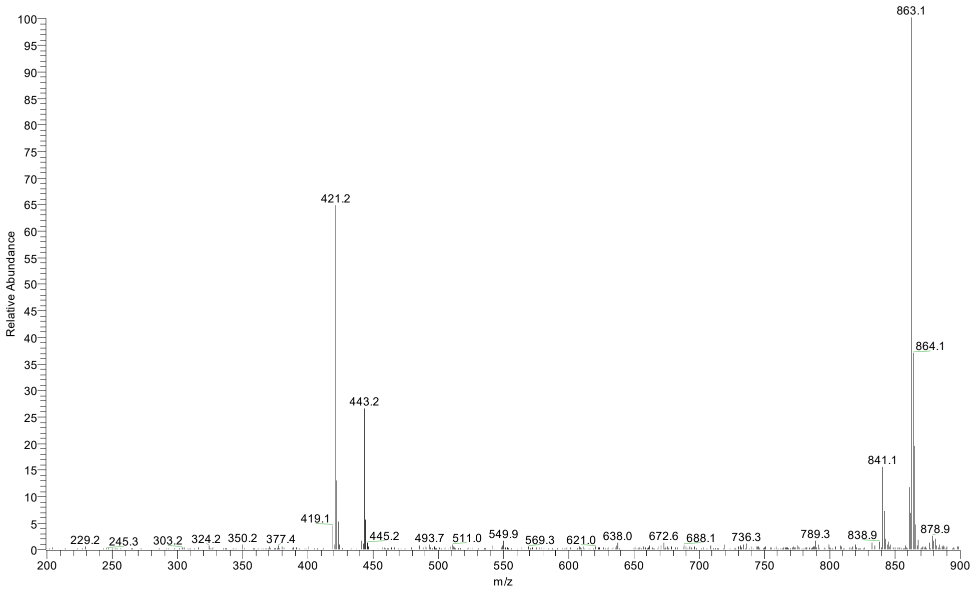

Figure S4. LCQ-MS spectrum of [Fe(L1+H$^+$)Cl]Cl.

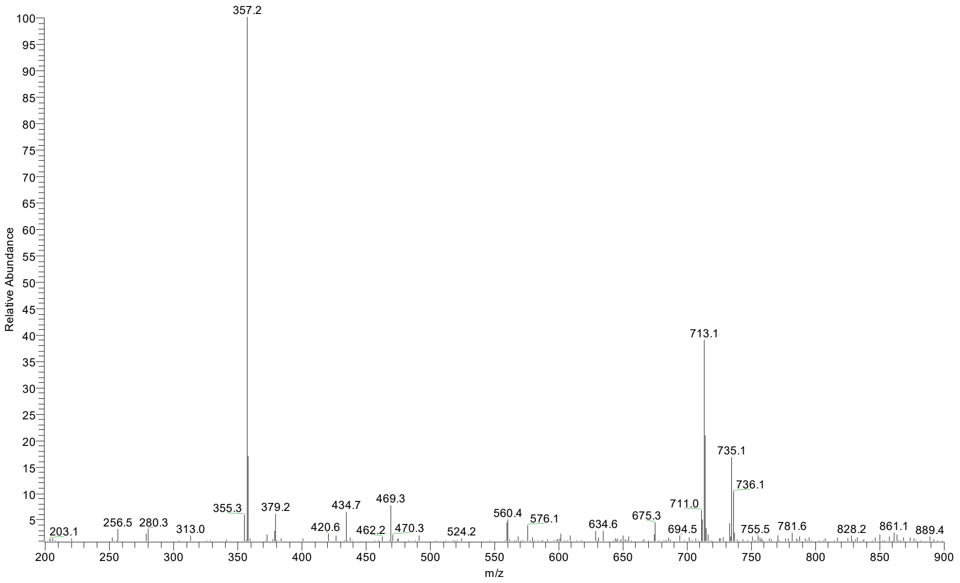

Figure S5. LCQ-MS spectrum of [Fe(L2)]Cl_2.

Table S1. Effective magnetic moment values through Evan's method for iron complexes.

Complex	μ_{eff}
Fe(L1)(OH_2)	5.8 ± 0.2
Fe(L2)	5.9 ± 0.2
Fe(L3)(OH_2)	5.9 ± 0.8

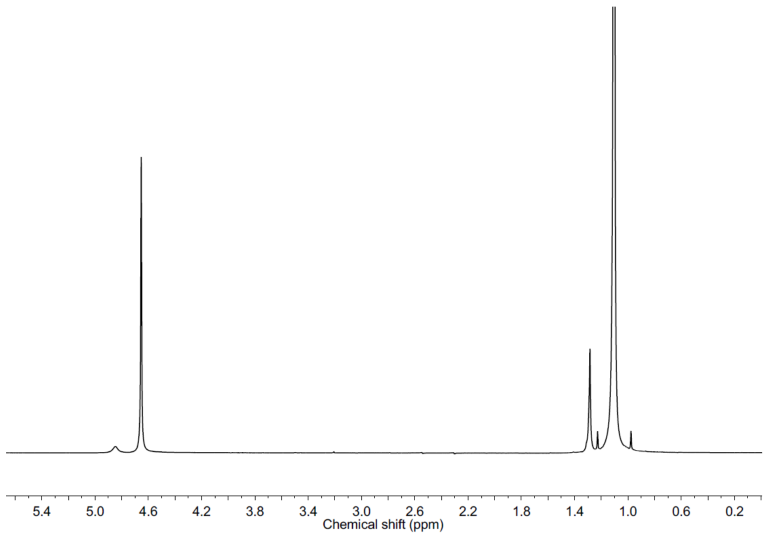

Figure S6. Evans Method NMR spectrum of 3.3 mM [Fe(L1 + H+)Cl]Cl in 5% t-butanol by volume D_2O.

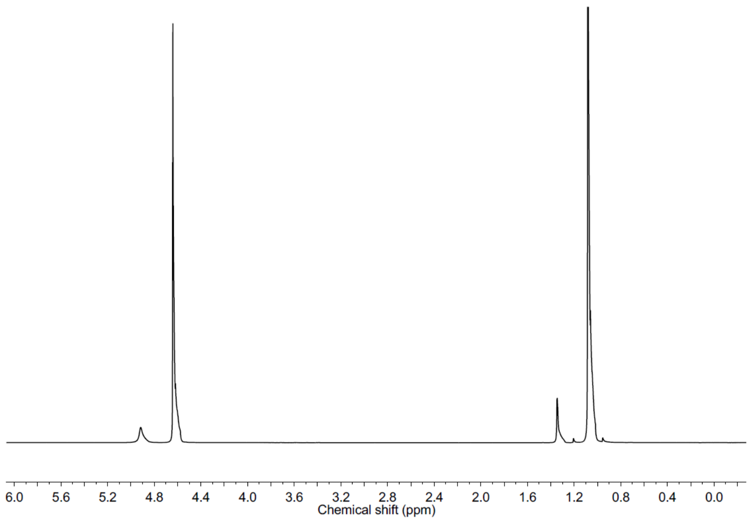

Figure S7. Evans Method NMR spectrum of 5 mM $[Fe(L2)]Cl_2$ in 5% t-butanol by volume D_2O.

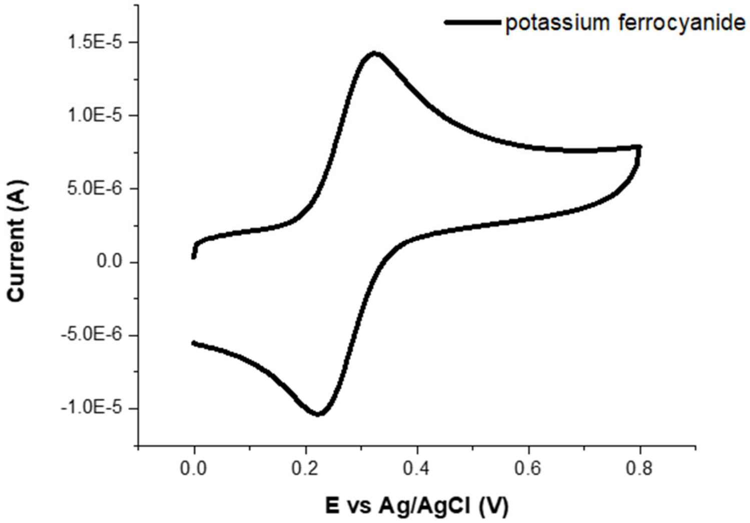

Figure S8. Cyclic voltammograms of 1.00 mM potassium ferricyanide solution in water with potassium chloride (0.100 M) as the supporting electrolyte and HEPES buffer. Full sweep widths were taken between -1.5V and 1.5V, at a scan rate of 100 mV/s [1]

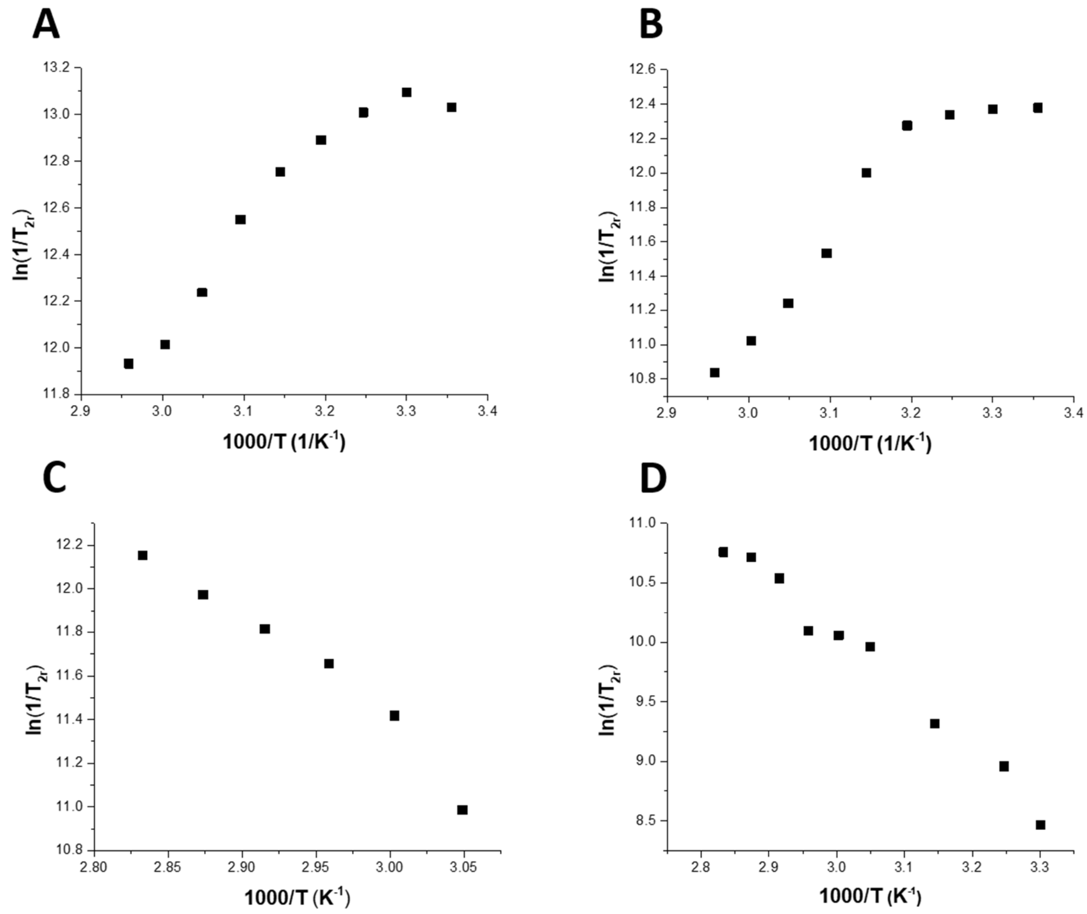

Figure S9. Reduced [17]O transverse relation rate constant ($\ln(1/T_{2r})$) as a function of temperature in solutions containing 10.0 mM Fe(L1)(OH_2) measured at pH 3.5 (A), at pH 4.3 (B), at pH 6.6 (C) and at pH 7.8 (D).

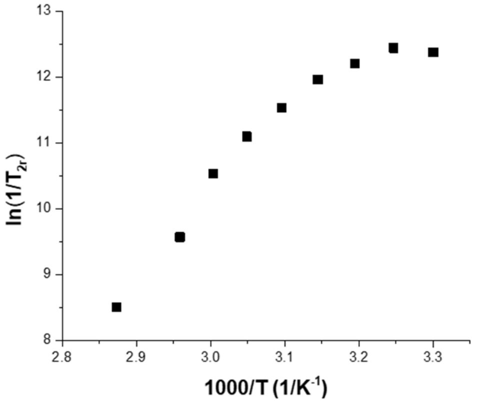

Figure S10. Reduced [17]O transverse relation rate constant ($\ln(1/T_{2r})$) as a function of temperature in solutions containing 10.0 mM Fe(L2) measured at pH 3.5.

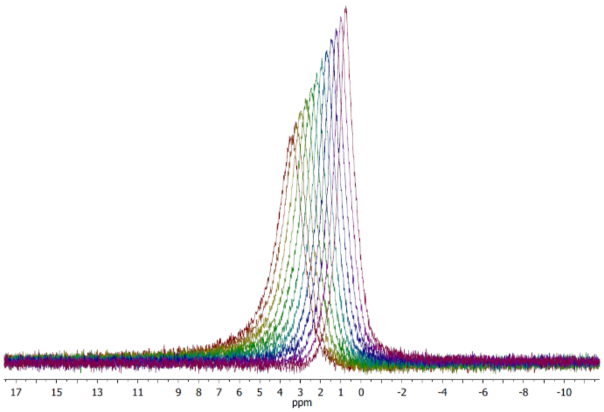

Figure S11. Shift and line broadening graphs of the ^{17}O resonance of 1% $H_2{}^{17}O$ standard solution. All graphs show a decrease in the chemical shift with increasing temperature.

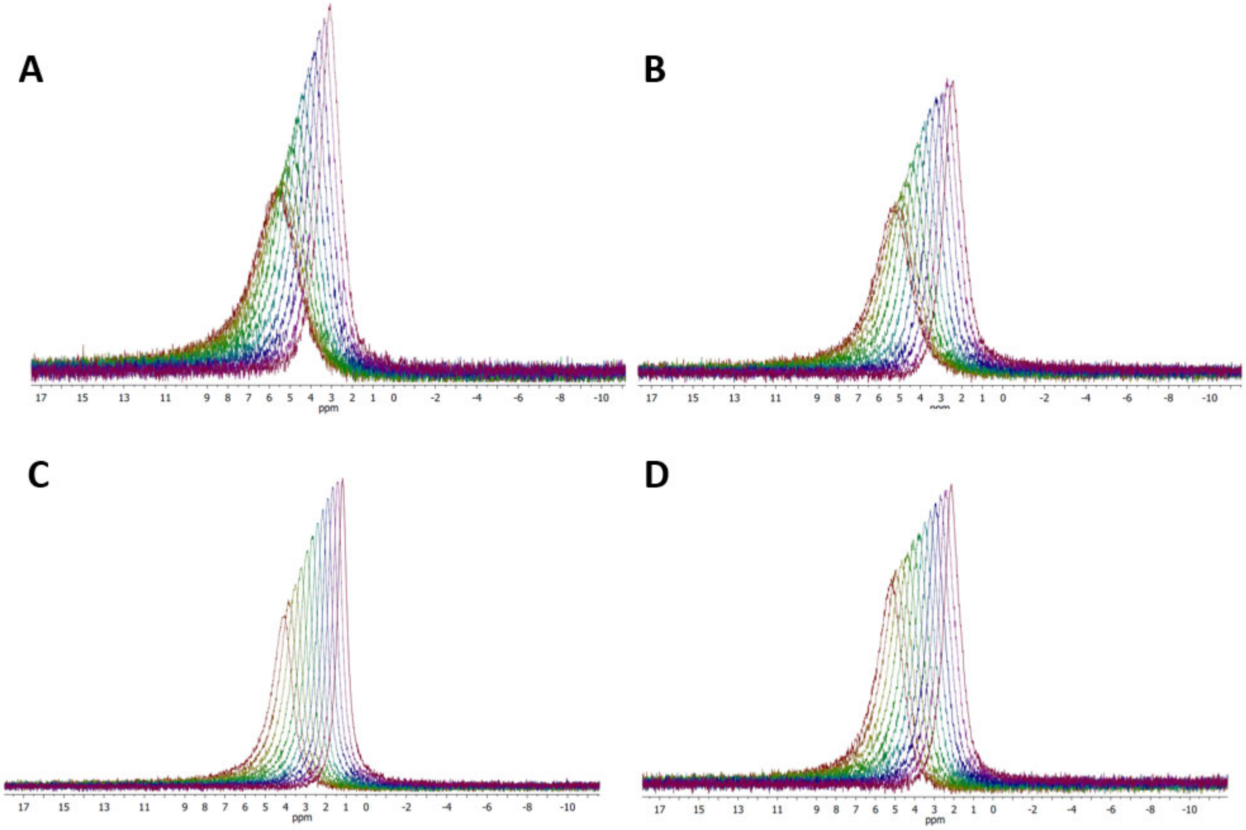

Figure S12. Shift and line broadening graphs of the ^{17}O resonance of 20 mM Fe(L1)(OH_2) solutions at variable temperatures at pH 3.5 (A), at pH 4.3 (B), at pH 6.6 (C) and at pH 7.8 (D).

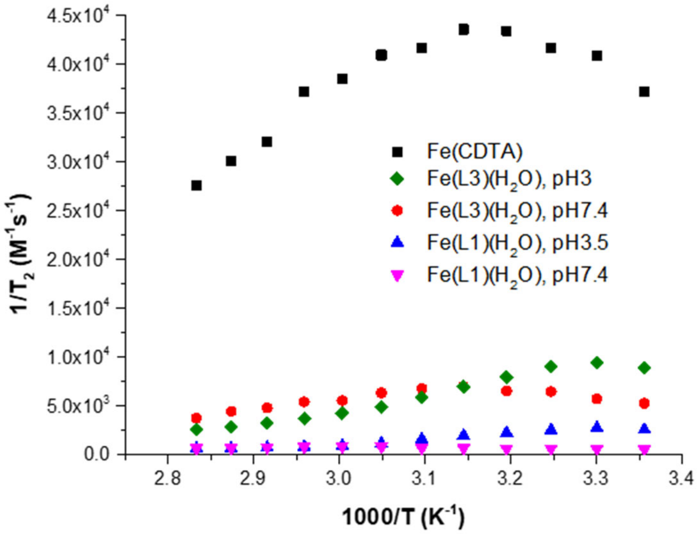

Figure S13. Comparison ^{17}O transverse relaxivity at pH 7.4 and at pH 3.5 as a function of temperature for Fe(L1)(OH_2), Fe(L3)(OH_2) and Fe(CDTA).

**Eq. S1**	$A + H^{+} \rightleftharpoons AH$	$\mathbf{K_1} = \dfrac{[\mathbf{AH}]}{[\mathbf{A}][\mathbf{H}^{+}]}$
**Eq. S2**	$AH + H^{+} \rightleftharpoons AH_2$	$\mathbf{K_2} = \dfrac{[\mathbf{AH_2}]}{[\mathbf{AH}][\mathbf{H}^{+}]}$

Equilibrium expressions and species definition.

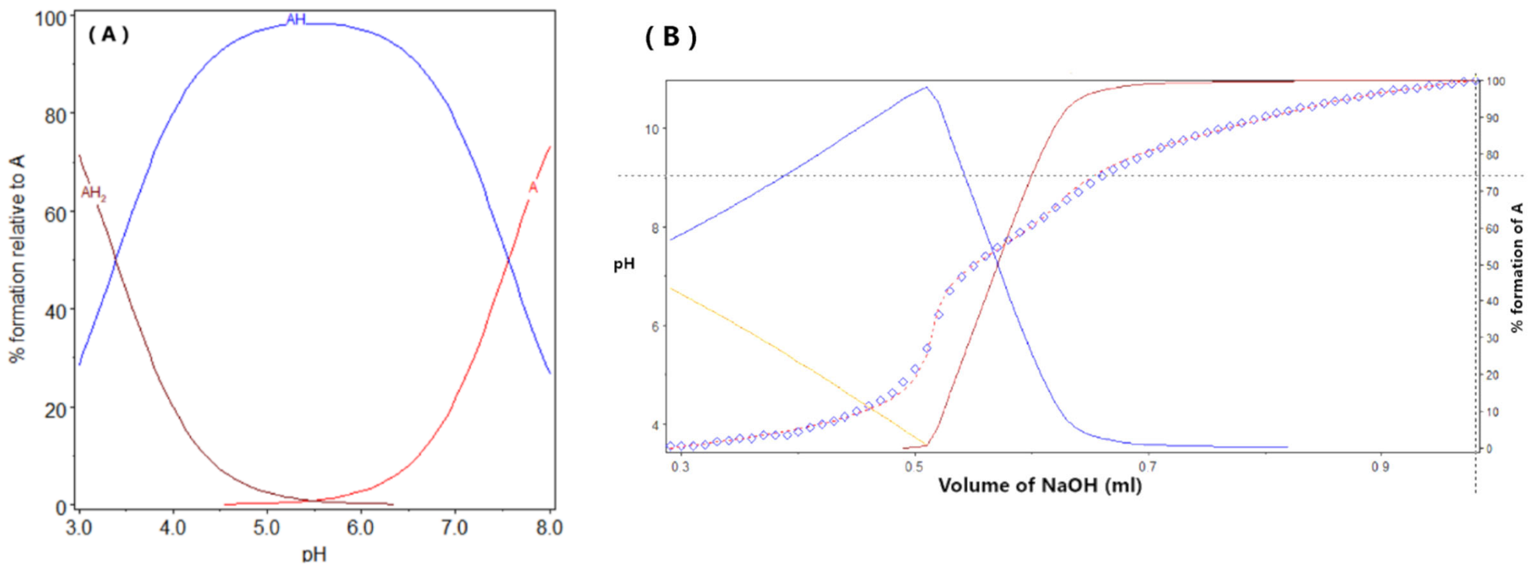

Figure S14. (A) Speciation diagram from pH potentiometric data of 0.527 mM Fe(L1)(OH_2)solution with 0.100 M NaCl and 1.00 mM meglumine at 25 °C. **(B)** The potentiometric titration curve with corresponding fit and equations to determine the equilibrium constants shown in **Table 1** for Fe(L1)(OH_2). The titrant concentration was [NaOH] = 0.09883 M. Here A is Fe(L1)(OH_2).

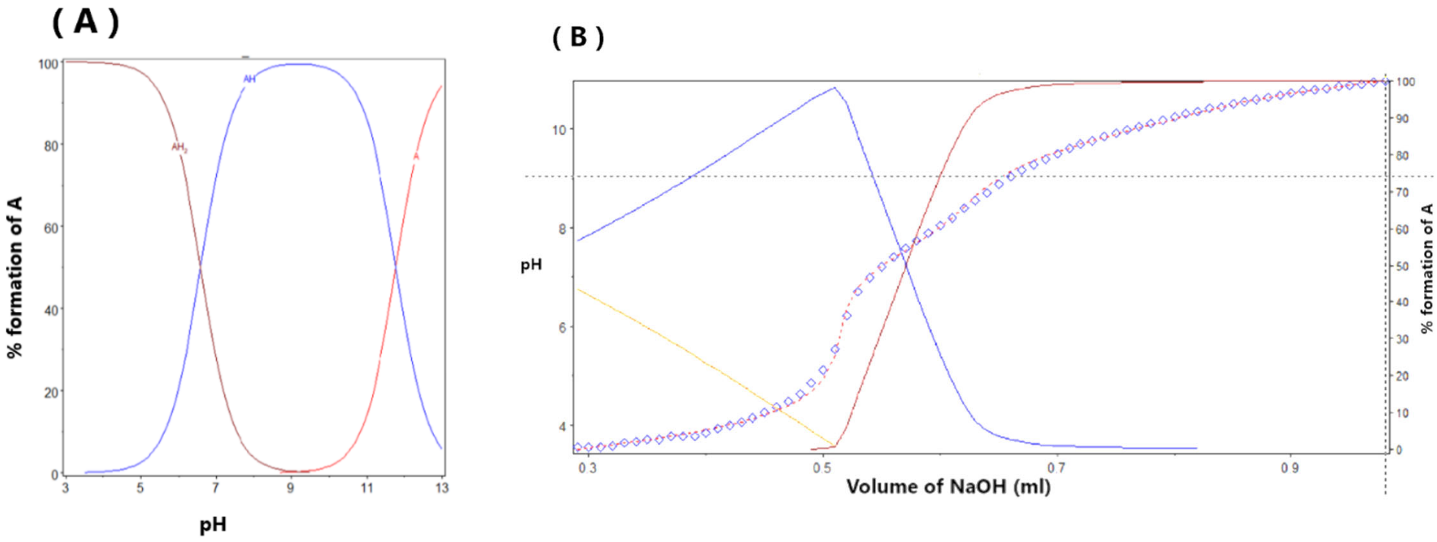

Figure S15. Speciation diagram from pH potentiometric data of 0.750 mM Fe(L2) solution with 0.100 M NaCl and 2.00 mM meglumine at 25 °C. Potentiometry titration curve with corresponding fit to determine the equilibrium constants shown in **Table 1** for Fe(L2). [NaOH] = 0.09486 M. Here A is Fe(L2-H^+).

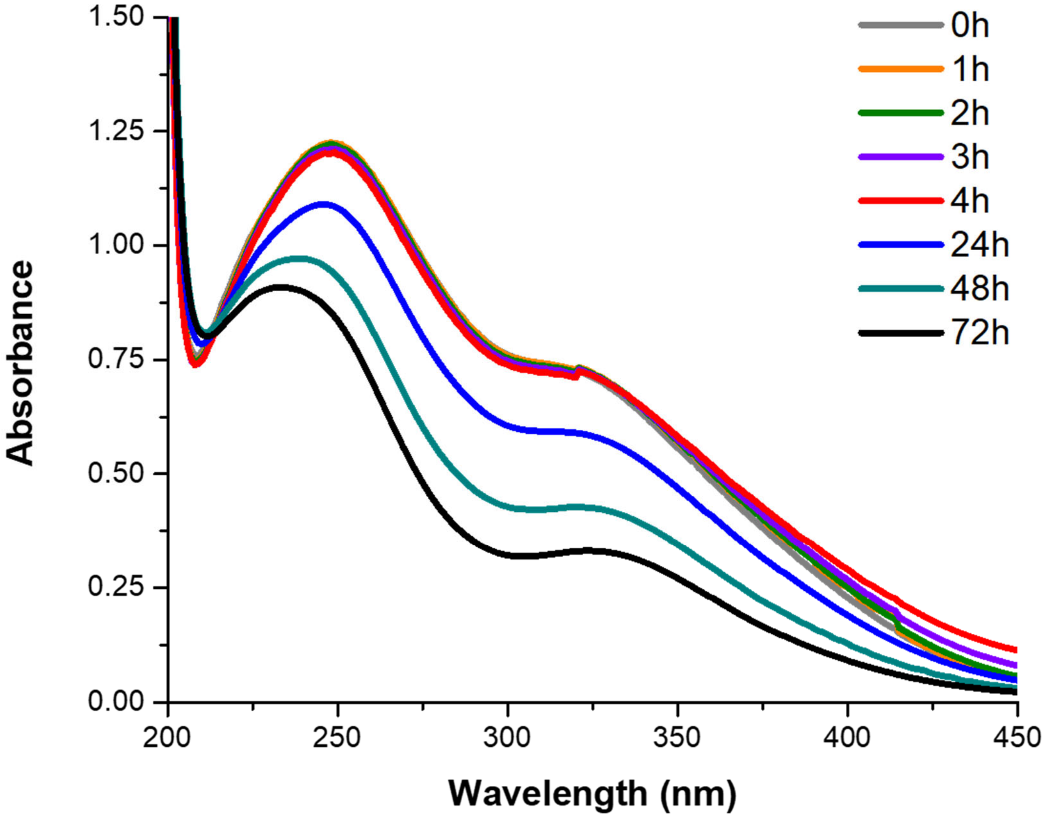

Figure S16. UV-vis absorbance spectra of $Fe(L1)(OH_2)$ were obtained over 72 hours at 37°C. Aqueous solutions contained 0.200 mM $Fe(L1)(OH_2)$ dissolved in 0.100 M HCl. Dissociation after 24 h was 18.1%. Dissociation after 72 h was 53.6%.

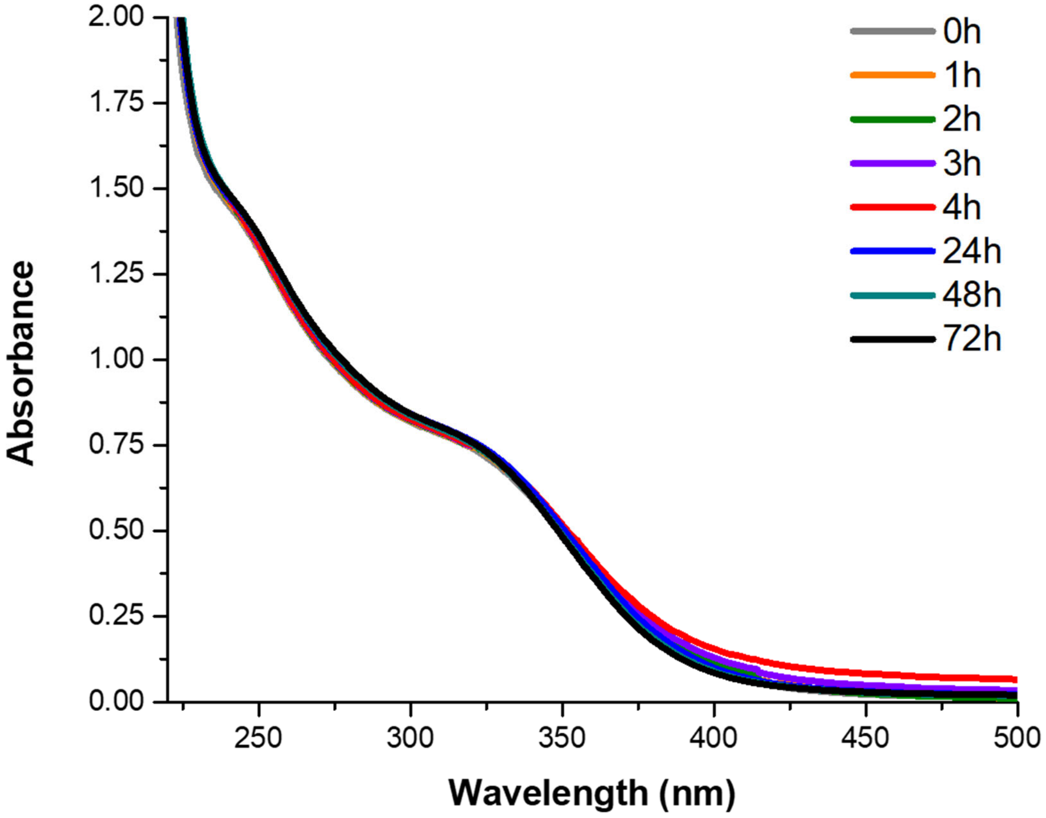

Figure S17. UV-vis absorbance spectra of Fe(L1)(OH_2) were obtained over 72 hours at 37°C. Aqueous solutions contained 0.200 mM Fe(L1)(OH_2) dissolved in 25.0 mM $NaHCO_3$, 0.500 mM Na_2HPO_4, 10 mM HEPES buffer at 7.4 pH.

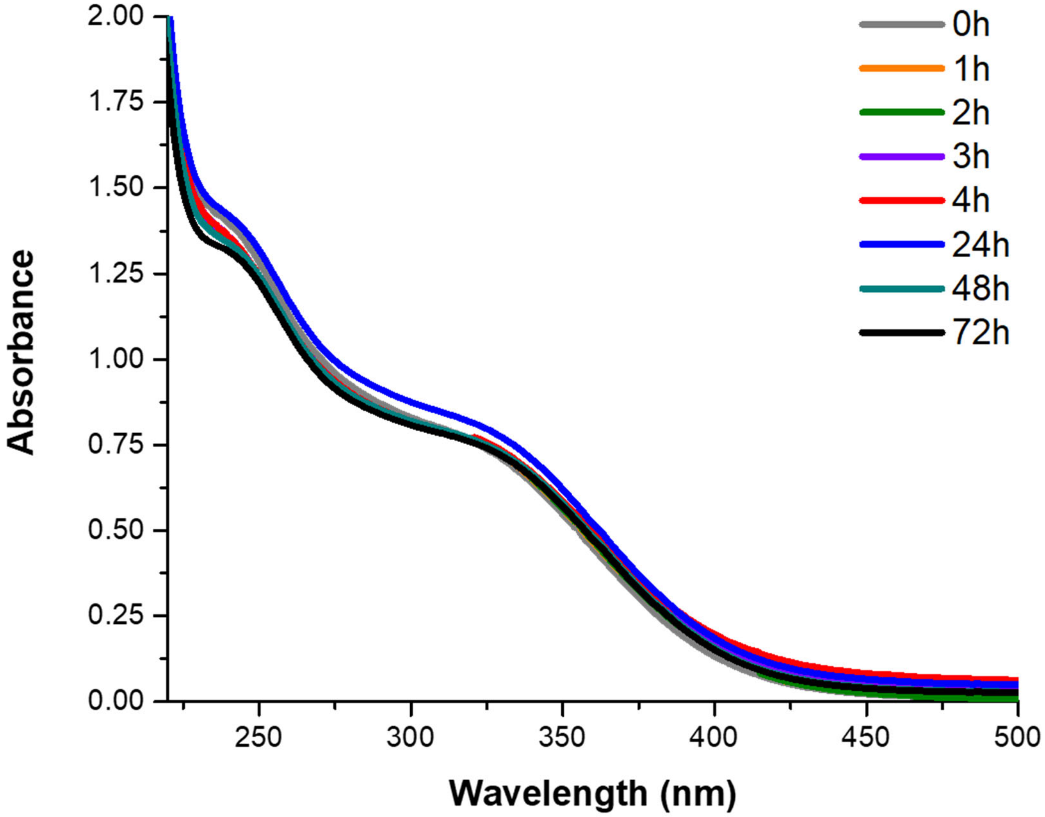

Figure S18. UV-vis absorbance spectra of Fe(L1)(OH_2)were obtained over 72 hours at 37°C. Aqueous solutions contained 0.200 mM Fe(L1)(OH_2) dissolved in 10.0 mM HEPES buffer at 7.4 pH. .

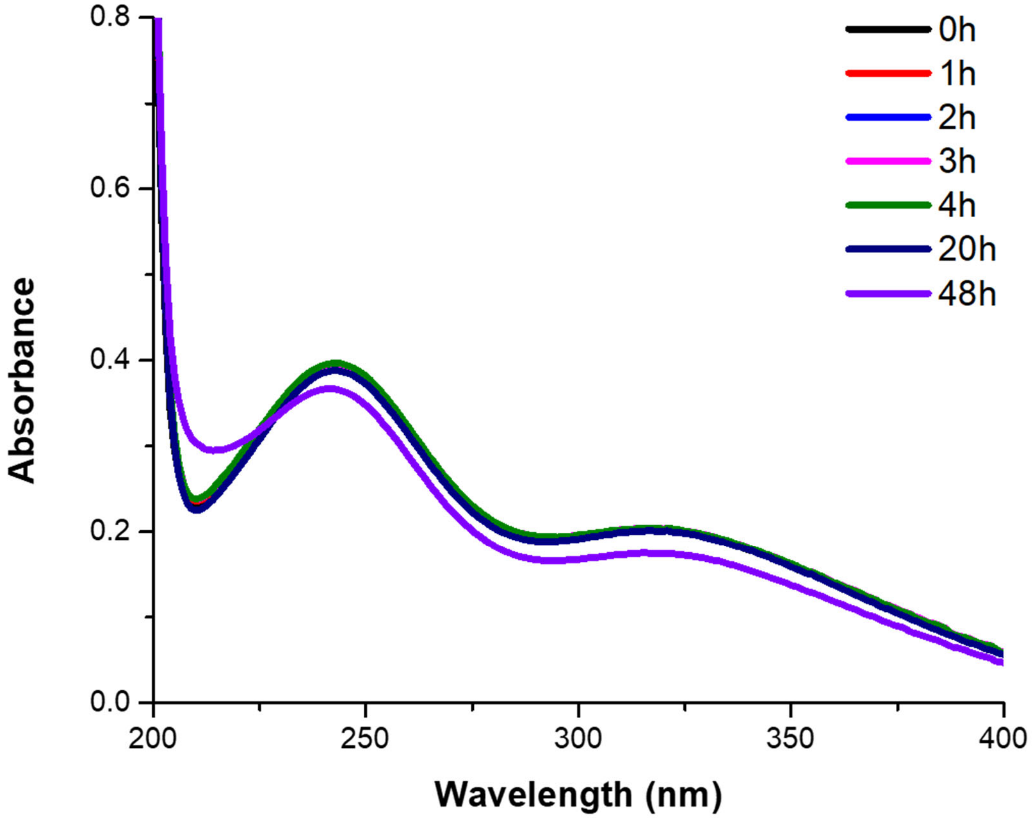

Figure S19. UV-vis absorbance spectra of Fe(L2) were obtained over 24 hours at 37 °C. Aqueous solutions contained 0.180 mM Fe(L2) dissolved in 0.100 M HCl. Dissociation after 48 h was 7.9 %.

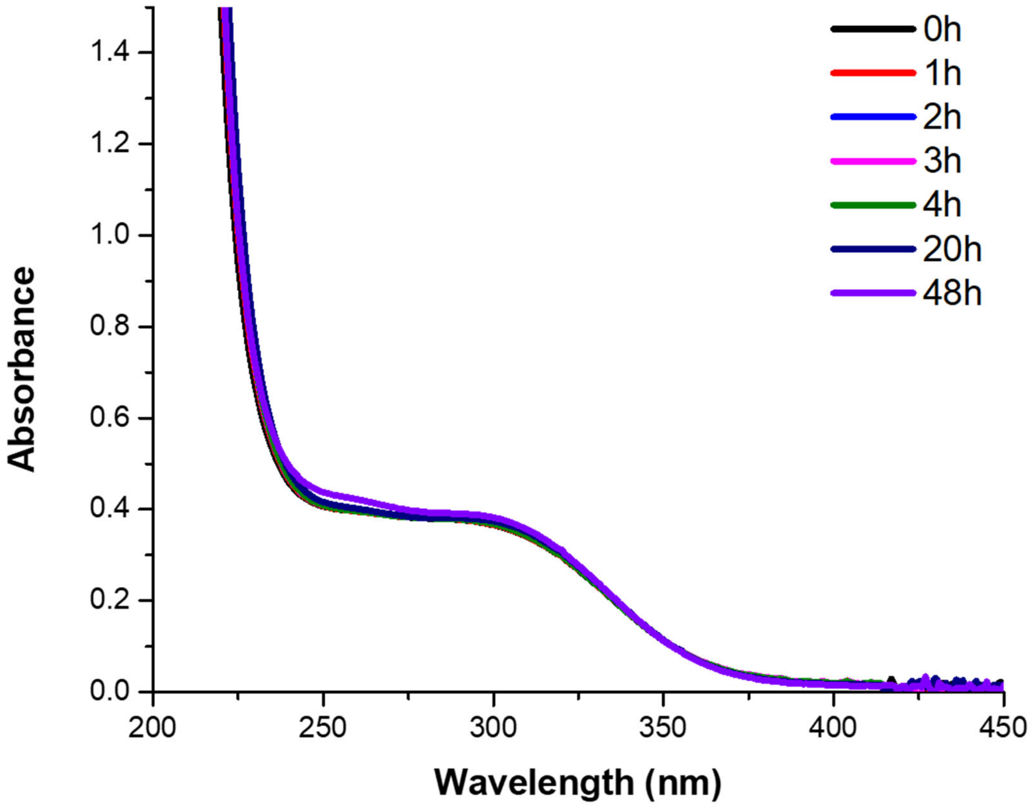

Figure S20. UV-vis absorbance spectra of Fe(L2) were obtained over 72 hours at 37 °C. Aqueous solutions contained 0.180 mM Fe(L2) dissolved in 25.0 mM $NaHCO_3$, 0.50 mM Na_2HPO_4, 10.0 mM HEPES buffer at 7.4 pH.

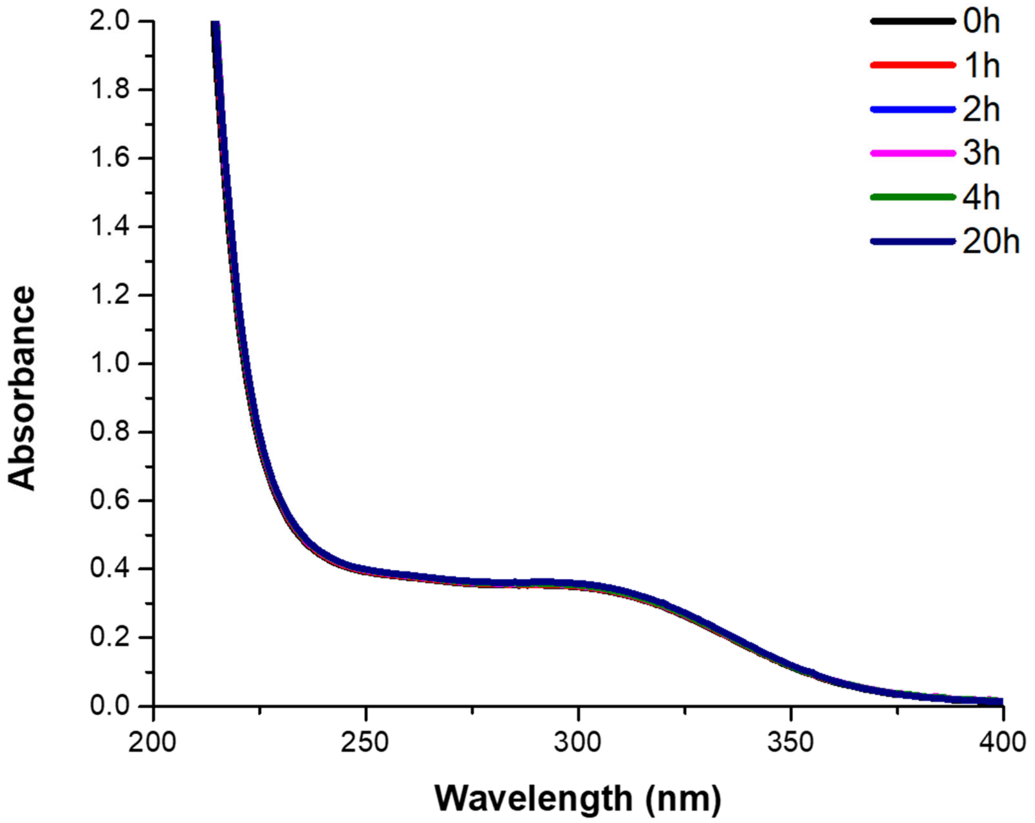

Figure S21. UV-vis absorbance spectra of Fe(L2) were obtained over 72 hours at 37 °C. Aqueous solutions contained 0.180 mM Fe(L2) dissolved in 10.0 mM HEPES buffer at 7.4 pH.

Table S2. The molar extinction coefficient (ε) values of the Fe complexes.

**Complex**	Ɛ(M^{-} cm^{-}) **245 nm**	Ɛ(M^{-} cm^{-}) **250 nm**	Ɛ(M^{-} cm^{-}) **300 nm**	Ɛ(M^{-} cm^{-}) **325 nm**	Ɛ(M^{-} cm^{-}) **330 nm**
Fe(L1)(OH_2)	6860	6097	----	3600	----
Fe(L2)	3160	----	2870	----	1570
Fe(L3)(OH_2)[a]	----	6980	----	----	3470

a. From reference [2]

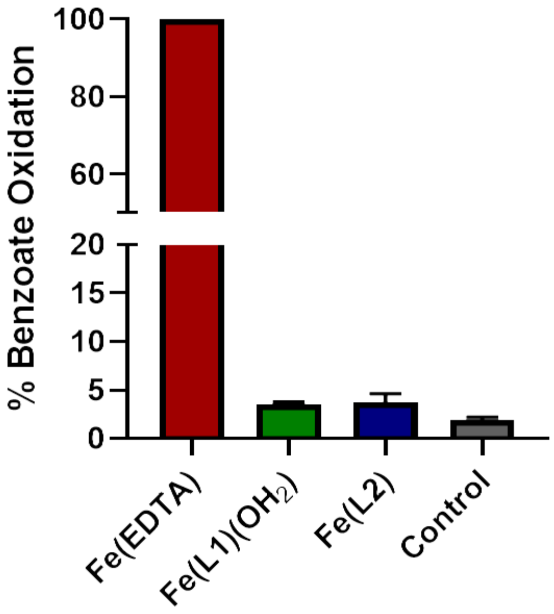

Figure S22. Percent benzoate oxidation by Fe(III) complexes normalized to amount produced by Fe(EDTA) complex.

Table S3. Percent benzoate oxidation by Fe(III) complexes normalized to amount produced by Fe(EDTA) complex. The solutions of Fe(III) complexes have 50.0 μM complex, 50.0 μM ascorbate, 50.0 μM benzoate, 50.0 μM H_2O_2, 0.100 M NaCl, 20.0 mM HEPES, pH 7.4 incubated at 37 °C for 1h. Control solution has same reagents without the iron complexes.

**Complex**	**% Benzoate Oxidation**
Fe(L1)(OH_2)	3.57 ± 0.18
Fe(L2)	3.76 ± 0.85
Fe(L3)(OH_2)[a]	4.00 ± 0.25
Control	1.92 ± 0.25

a. From reference [2]

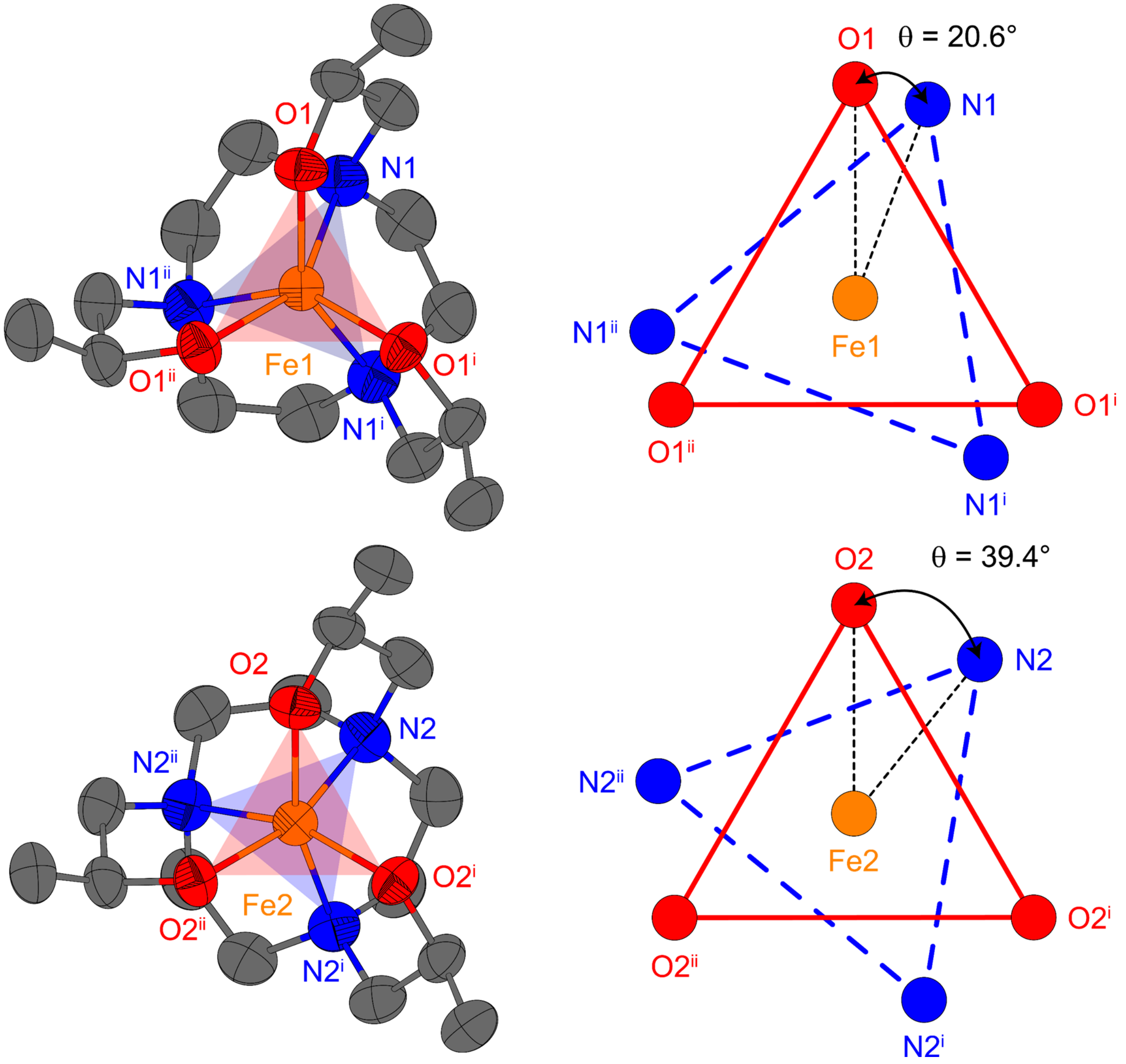

Figure S23. Left: ORTEP of the two Fe(L2) monomers and their respective mean planes with thermal ellipsoids drawn that the 50% probability level. $[FeCl_4]^-$ anions and hydrogen atoms were omitted for clarity. Right: Simplified illustration of the two monomers and their mean planes with their calculated twist angles. Symmetry Codes: (i) 1-y, 1+x, z; (ii) y-x, 1-x, z.

Table S4. Crystal data and structure refinement for Fe(L2).

Empirical formula	$C_{30}H_{63}Cl_{12}Fe_5N_6O_6$
Formula weight	1308.51
Temperature/K	90
Crystal system	trigonal
Space group	*R*32
a/Å	11.4070(2)
b/Å	11.4070(2)
c/Å	72.2652(18)
α/°	90
β/°	90
γ/°	120
Volume/Å^3	8143.3(4)
Z	6
ρ_{calc}g/cm^3	1.601
μ/mm^{-1}	1.937
F(000)	4002.0
Crystal size/mm^3	0.1 × 0.06 × 0.06
Radiation	MoK*α* (*λ* = 0.71073)
2Θ range for data collection/°	3.382 to 56.588
Index ranges	-15 ≤ h ≤ 15, -15 ≤ k ≤ 15, -94 ≤ l ≤ 95
Reflections collected	56538
Independent reflections	4433 [R_{int} = 0.0401, R_{sigma} = 0.0220]
Data/restraints/parameters	4433/2/230
Goodness-of-fit on F^2	1.002
Final R indexes [I>=2σ (I)]	R_1 = 0.0314, wR_2 = 0.0722
Final R indexes [all data]	R_1 = 0.0563, wR_2 = 0.0821
Largest diff. peak/hole / e Å^{-3}	0.46/-0.34
Flack parameter	-0.012(9)

Table S5. Selected Bond Lengths for Fe(L2).

Atom	Atom	Length (Å)
Fe1	O1	1.967(2)
Fe1	N1	2.188(3)
Fe2	O2	1.949(2)
Fe2	N2	2.176(3)

Table S6. Selected Bond Angles for Fe(L2).

Atom	Atom	Atom	Angle (°)
O1	Fe1	N1	77.6(1)
O2	Fe2	N2	80.1(1)

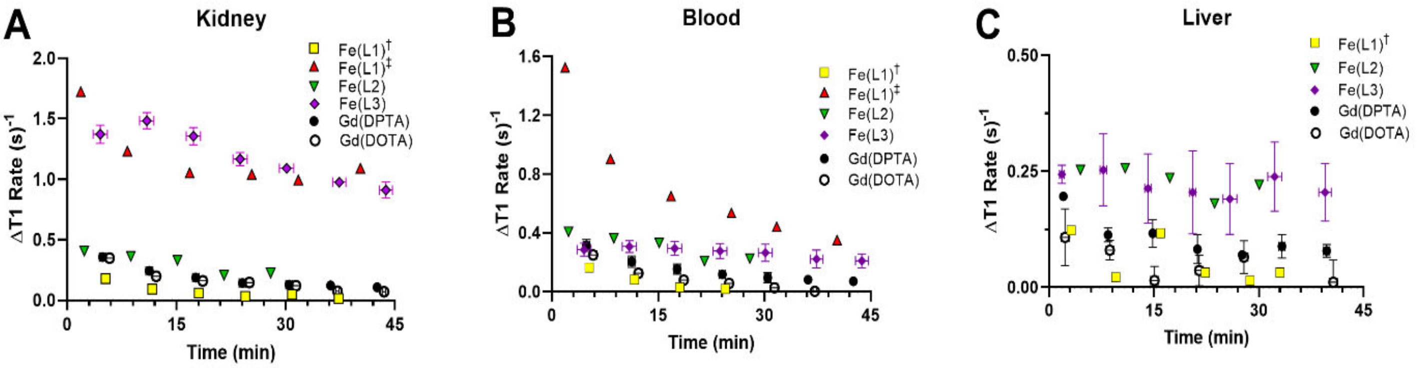

Figure S24. Changes in T_1 rates for Fe(L1)(OH_2) († is 0.050 mmol/kg dose); (‡ is 0.200 mmol/kg dose) Fe(L2), Fe(L3)(OH_2), Gd(DTPA) and Gd(DOTA) over time in the kidneys (A), inferior vena cava (B) and liver in healthy Balb/C mice at 4.7 T. A 0.050 mmol/kg dose† was used for all compounds, as well as a 0.200 mmol/kg dose‡ for Fe(L1). Liver data for Fe(L1)(OH_2) at 0.200 mmol/kg is not included due to an uncorrectable banding artifact often associated with TrueFISP acquisitions.

References

1. Radhi, M.; Al-Mulla, E.A.J.; Tan, W. Electrochemical characterization of the redox couple of Fe (III)/Fe (II) mediated by grafted polymer electrode. *Res. Chem. Intermed.* **2014**, *40*, 179-192.

2. Snyder, E.M.; Asik, D.; Abozeid, S.M.; Burgio, A.; Bateman, G.; Turowski, S.G.; Spernyak, J.; Morrow, J.R. A new class of Fe (III) macrocyclic complexes with alcohol donor groups as effective T1 MRI contrast agents. *Angew. Chem. Int. Ed.* **2019**, 59, 2414-2419.
